# Global pattern, trend, and cross-country inequality of early musculoskeletal disorders from 1990 to 2019, with projection from 2020 to 2050

**DOI:** 10.1016/j.medj.2024.04.009

**Published:** 2024-08-09

**Authors:** GBD 2019 MSK in Adolescents Collaborators, Yingzhao Jin, Cui Guo, Mohammadreza Abbasian, Mitra Abbasifard, J. Haxby Abbott, Auwal Abdullahi, Aidin Abedi, Hassan Abidi, Hassan Abolhassani, Eman Abu-Gharbieh, Salahdein Aburuz, Ahmed Abu-Zaid, Isaac Yeboah Addo, Oyelola A. Adegboye, Abiola Victor Adepoju, Wirawan Adikusuma, Qorinah Estiningtyas Sakilah Adnani, Shahin Aghamiri, Danish Ahmad, Ayman Ahmed, Janardhana P. Aithala, Shiva Akhlaghi, Sreelatha Akkala, Tariq A. Alalwan, Mohammed Albashtawy, Hediyeh Alemi, Fadwa Alhalaiqa Naji Alhalaiqa, Endale Alemayehu Ali, Sami Almustanyir, Rajaa M. Al-Raddadi, Nelson J. Alvis-Zakzuk, Yaser Mohammed Al-Worafi, Hosam Alzahrani, Karem H. Alzoubi, Sohrab Amiri, Hubert Amu, Jimoh Amzat, David B. Anderson, Abhishek Anil, Benny Antony, Jalal Arabloo, Damelash Areda, Al Artaman, Anton A. Artamonov, Krishna K. Aryal, Mohammad Asghari-Jafarabadi, Tahira Ashraf, Seyyed Shamsadin Athari, Bantalem Tilaye Atinafu, Maha Moh’d Wahbi Atout, Sina Azadnajafabad, Hamed Azhdari Tehrani, Ahmed Y. Azzam, Alaa Badawi, Nayereh Baghcheghi, Ruhai Bai, Vali Baigi, Maciej Banach, Morteza Banakar, Biswajit Banik, Mainak Bardhan, Till Winfried Bärnighausen, Hiba Jawdat Barqawi, Amadou Barrow, Azadeh Bashiri, Kavita Batra, Mojtaba Bayani, Nebiyou Simegnew Bayileyegn, Ahmet Begde, Kebede A. Beyene, Akshaya Srikanth Bhagavathula, Pankaj Bhardwaj, Gurjit Kaur Bhatti, Jasvinder Singh Bhatti, Rajbir Bhatti, Ali Bijani, Veera R. Bitra, Javier Brazo-Sayavera, Rachelle Buchbinder, Katrin Burkart, Yasser Bustanji, Muhammad Hammad Butt, Luis Alberto Cámera, Felix Carvalho, Vijay Kumar Chattu, Akhilanand Chaurasia, Guangjin Chen, Haowei Chen, Lingxiao Chen, Steffan Wittrup McPhee Christensen, Dinh-Toi Chu, Isaac Sunday Chukwu, Josielli Comachio, Natália Cruz-Martins, Sarah Cuschieri, Sriharsha Dadana, Omid Dadras, Xiaochen Dai, Zhaoli Dai, Saswati Das, Mohsen Dashti, Ivan Delgado-Enciso, Biniyam Demisse, Edgar Denova-Gutiérrez, Belay Desye, Syed Masudur Rahman Dewan, Sameer Dhingra, Mengistie Diress, Thanh Chi Do, Thao Huynh Phuong Do, Khanh Duy Khanh Doan, Sulagna Dutta, Arkadiusz Marian Dziedzic, Hisham Atan Edinur, Michael Ekholuenetale, Muhammed Elhadi, Sharareh Eskandarieh, Francesco Esposito, Adeniyi Francis Fagbamigbe, Parisa Farokh, Ali Fatehizadeh, Alireza Feizkhah, Ginenus Fekadu, Nuno Ferreira, Getahun Fetensa, Florian Fischer, Behzad Foroutan, Masoumeh Foroutan Koudehi, Richard Charles Franklin, Takeshi Fukumoto, Aravind P. Gandhi, Balasankar Ganesan, Shuo-Yan Gau, Rupesh K. Gautam, Abadi Kahsu Gebre, Miglas W.W. Gebregergis, Bardiya Ghaderi Yazdi, Ali Gholami, Tiffany K. Gill, Pouya Goleij, Mansueto Gomes-Neto, Anmol Goyal, Simon Matthew Graham, Bin Guan, Bhawna Gupta, Indarchand Ratanlal Gupta, Sapna Gupta, Veer Bala Gupta, Vivek Kumar Gupta, Farrokh Habibzadeh, Wase Benti Hailu, Ramtin Hajibeygi, Rabih Halwani, Josep Maria Haro, Jan Hartvigsen, Ahmed I. Hasaballah, Johannes Haubold, Jeffrey J. Hebert, Mohamed I. Hegazy, Golnaz Heidari, Mohammad Heidari, Kamal Hezam, Yuta Hiraike, Hassan Hosseinzadeh, Mehdi Hosseinzadeh, Amir Human Hoveidaei, Chi-Jen Hsu, Md Nazmul Huda, Hong-Han Huynh, Bing-Fang Hwang, Segun Emmanuel Ibitoye, Adalia I. Ikiroma, Irena M. Ilic, Milena D. Ilic, Arad Iranmehr, Sheikh Mohammed Shariful Islam, Nahlah Elkudssiah Ismail, Hiroyasu Iso, Masao Iwagami, Assefa N. Iyasu, Louis Jacob, Abdollah Jafarzadeh, Kasra Jahankhani, Nityanand Jain, Ammar Abdulrahman Jairoun, Balamurugan Janakiraman, Umesh Jayarajah, Shubha Jayaram, Jayakumar Jeganathan, Mohammad Jokar, Jost B. Jonas, Tamas Joo, Nitin Joseph, Charity Ehimwenma Joshua, Gebisa Guyasa Kabito, Vineet Kumar Kamal, Himal Kandel, Rami S. Kantar, Jafar Karami, Ibraheem M. Karaye, Arman Karimi Behnagh, Navjot Kaur, Foad Kazemi, Shemsu Kedir, Mohamad Mehdi Khadembashiri, Mohammad Amin Khadembashiri, Yousef Saleh Khader, Himanshu Khajuria, Mohammad Jobair Khan, Moien AB Khan, Mahammed Ziauddin Khan Suheb, Haitham Khatatbeh, Moawiah Mohammad Khatatbeh, Sorour Khateri, Hamid Reza Khayat Kashani, Mohammad Saeid Khonji, Jagdish Khubchandani, Saeid Kian, Adnan Kisa, Aiggan Tamene Kitila, Ali-Asghar Kolahi, Hamid Reza Koohestani, Oleksii Korzh, Karel Kostev, Ashwin Laxmikant Kotnis, Ai Koyanagi, Kewal Krishan, Mohammed Kuddus, Narinder Kumar, Maria Dyah Kurniasari, Muhammad Awwal Ladan, Chandrakant Lahariya, Tri Laksono, Tea Lallukka, Iván Landires, Savita Lasrado, Basira Kankia Lawal, Thao Thi Thu Le, Trang Diep Thanh Le, Munjae Lee, Wei-Chen Lee, Yo Han Lee, Temesgen L. Lerango, David Lim, Stephen S. Lim, Giancarlo Lucchetti, Zheng Feei Ma, Azzam A. Maghazachi, Nastaran Maghbouli, Elaheh Malakan Rad, Armaan Malhotra, Ahmad Azam Malik, Mohammad Ali Mansournia, Lorenzo Giovanni Mantovani, Emmanuel Manu, Yasith Mathangasinghe, Antonio Mazzotti, Steven M. McPhail, Belayneh Mengist, Mohamed Kamal Mesregah, Tomislav Mestrovic, Ted R. Miller, Le Huu Nhat Minh, Mohammad Mirahmadi Eraghi, Erkin M. Mirrakhimov, Awoke Misganaw, Hashem Mohamadian, Ashraf Mohamadkhani, Nouh Saad Mohamed, Esmaeil Mohammadi, Soheil Mohammadi, Mesud Mohammed, Hoda Mojiri-Forushani, Ali H. Mokdad, Kaveh Momenzadeh, Sara Momtazmanesh, Lorenzo Monasta, Fateme Montazeri, Yousef Moradi, Shane Douglas Morrison, Ebrahim Mostafavi, Parsa Mousavi, Seyed Ehsan Mousavi, Admir Mulita, Efrén Murillo-Zamora, Ghulam Mustafa, Sathish Muthu, Ganesh R. Naik, Mukhammad David Naimzada, Noureddin Nakhostin Ansari, Sreenivas Narasimha Swamy, Shumaila Nargus, Paulo R.C. Nascimento, Amirreza Naseri, Zuhair S. Natto, Muhammad Naveed, Biswa Prakash Nayak, Athare Nazri-Panjaki, Mohammad Negaresh, Hadush Negash, Seyed Aria Nejadghaderi, Dang H. Nguyen, Hau Thi Hien Nguyen, Hien Quang Nguyen, Phat Tuan Nguyen, Van Thanh Nguyen, Robina Khan Niazi, Akinyemi O.D. Ofakunrin, Hassan Okati-Aliabad, Osaretin Christabel Okonji, Matthew Idowu Olatubi, Mohammad Mehdi Ommati, Michal Ordak, Mayowa O. Owolabi, Mahesh P A, Jagadish Rao Padubidri, Feng Pan, Ioannis Pantazopoulos, Seoyeon Park, Jay Patel, Shankargouda Patil, Shrikant Pawar, Paolo Pedersini, Prince Peprah, Simone Perna, Ionela-Roxana Petcu, Fanny Emily Petermann-Rocha, Hoang Tran Pham, Manon Pigeolet, Elton Junio Sady Prates, Fakher Rahim, Zahra Rahimi, Shahram Rahimi-Dehgolan, Vafa Rahimi-Movaghar, Mohammad Hifz Ur Rahman, Masoud Rahmati, Shakthi Kumaran Ramasamy, Premkumar Ramasubramani, Deepthi Rapaka, Sina Rashedi, Vahid Rashedi, Mohammad-Mahdi Rashidi, Ashkan Rasouli-Saravani, Salman Rawaf, Murali Mohan Rama Krishna Reddy, Elrashdy Moustafa Mohamed Redwan, Nazila Rezaei, Negar Rezaei, Nima Rezaei, Zahed Rezaei, Abanoub Riad, Leonardo Roever, Sharareh Roshanzamir, Priyanka Roy, Guilherme de Andrade Ruela, Aly M.A. Saad, Basema Saddik, Farideh Sadeghian, Umar Saeed, Azam Safary, Amene Saghazadeh, Dominic Sagoe, Fatemeh Saheb Sharif-Askari, Narjes Saheb Sharif-Askari, Amirhossein Sahebkar, Joseph W. Sakshaug, Afeez Abolarinwa Salami, Mohamed A. Saleh, Sana Salehi, Sara Samadzadeh, Yoseph Leonardo Samodra, Vijaya Paul Samuel, Djanilson B. Santos, Milena M. Santric-Milicevic, Muhammad Arif Nadeem Saqib, Aswini Saravanan, Susan Sawyer, Benedikt Michael Schaarschmidt, Sabyasachi Senapati, Yashendra Sethi, Allen Seylani, Amir Shafaat, Mahan Shafie, Saeed Shahabi, Ataollah Shahbandi, Shayan Shahrokhi, Masood Ali Shaikh, Muhammad Aaqib Shamim, Mohammad Ali Shamshirgaran, Sadaf Sharfaei, Amin Sharifan, Azam Sharifi, Rajendra Sharma, Saurab Sharma, Bereket Beyene Shashamo, Linhong Shi, Mika Shigematsu, Rahman Shiri, Velizar Shivarov, Emmanuel Edwar Siddig, Ehsan Sinaei, Ambrish Singh, Jasvinder A. Singh, Paramdeep Singh, Surjit Singh, Shweta Singla, Md Shahjahan Siraj, Anna Aleksandrovna Skryabina, Ranjan Solanki, Yonatan Solomon, Antonina V. Starodubova, Chandan Kumar Swain, Stella Talic, Nathan Y. Tat, Mohamad-Hani Temsah, Dufera Rikitu Terefa, Riki Tesler, Rekha Thapar, Samar Tharwat, Rasiah Thayakaran, Jansje Henny Vera Ticoalu, Marcos Roberto Tovani-Palone, Biruk Shalmeno Tusa, Sree Sudha Ty, Aniefiok John Udoakang, Seyed Mohammad Vahabi, Rohollah Valizadeh, Jef Van den Eynde, Shoban Babu Varthya, Tommi Juhani Vasankari, Narayanaswamy Venketasubramanian, Jorge Hugo Villafañe, Vasily Vlassov, Anh Truc Vo, Linh Gia Vu, Yuan-Pang Wang, Taweewat Wiangkham, Nuwan Darshana Wickramasinghe, Andrea Sylvia Winkler, Ai-Min Wu, Ali Yadollahpour, Galal Yahya, Naohiro Yonemoto, Yuyi You, Mustafa Z. Younis, Fathiah Zakham, Moein Zangiabadian, Armin Zarrintan, Chenwen Zhong, Hengxing Zhou, Zhaochen Zhu, Magdalena Zielińska, Yossef Teshome Zikarg, Osama A. Zitoun, Mohammad Zoladl, Lai-Shan Tam, Dongze Wu

**Keywords:** musculoskeletal disorders, Global Burden of Disease study, trend, inequality, pattern, rheumatoid arthritis, osteoarthritis, gout, low back pain, neck pain

## Abstract

**Background:**

This study aims to estimate the burden, trends, forecasts, and disparities of early musculoskeletal (MSK) disorders among individuals ages 15 to 39 years.

**Methods:**

The global prevalence, years lived with disabilities (YLDs), disability-adjusted life years (DALYs), projection, and inequality were estimated for early MSK diseases, including rheumatoid arthritis (RA), osteoarthritis (OA), low back pain (LBP), neck pain (NP), gout, and other MSK diseases (OMSKDs).

**Findings:**

More adolescents and young adults were expected to develop MSK disorders by 2050. Across five age groups, the rates of prevalence, YLDs, and DALYs for RA, NP, LBP, gout, and OMSKDs sharply increased from ages 15–19 to 35–39; however, these were negligible for OA before age 30 but increased notably at ages 30–34, rising at least 6-fold by 35–39. The disease burden of gout, LBP, and OA attributable to high BMI and gout attributable to kidney dysfunction increased, while the contribution of smoking to LBP and RA and occupational ergonomic factors to LBP decreased. Between 1990 and 2019, the slope index of inequality increased for six MSK disorders, and the relative concentration index increased for gout, NP, OA, and OMSKDs but decreased for LBP and RA.

**Conclusions:**

Multilevel interventions should be initiated to prevent disease burden related to RA, NP, LBP, gout, and OMSKDs among individuals ages 15–19 and to OA among individuals ages 30–34 to tightly control high BMI and kidney dysfunction.

**Funding:**

The Global Burden of Disease study is funded by the Bill and Melinda Gates Foundation. The project is funded by the Scientific Research Fund of Sichuan Academy of Medical Sciences & Sichuan Provincial People’s Hospital (2022QN38).

## Highlights

• More adolescents and young adults (AYAs) are expected to develop MSK by 2050
• There are increasing contributions of high BMI to gout, LBP, and OA and of KD to gout
• The slope index of inequality increased for six musculoskeletal disorders
• More attention to AYAs is needed to bring targeted risk factors under control

## Context and significance

Adolescence and young adulthood are life phases in which the opportunities for health are great and future patterns of health are established. A focus on musculoskeletal disorders among individuals ages 15 to 39 (early MSK disorders) is central to managing the disease burden of MSK disease in the era of an aging population. Researchers from GBD 2019 MSK in Adolescents Collaborators have shown (1) a rising disease burden of early MSK disease by 2050; (2) increasing contributions of high body mass index to gout, low back pain, and osteoarthritis and increasing contributions of kidney dysfunction to gout; and (3) an enlarging slope index of inequality for six MSK disorders and relative concentration index for gout, neck pain, osteoarthritis, and other MSK disorders. The authors highlight that multilevel interventions should be started to prevent the disease burden of early MSK disorders at the respective optimal ages and to decrease the contributions of high body mass index and kidney dysfunction to the disease burden of early MSK diseases.

## Graphical abstract

**Figure:**
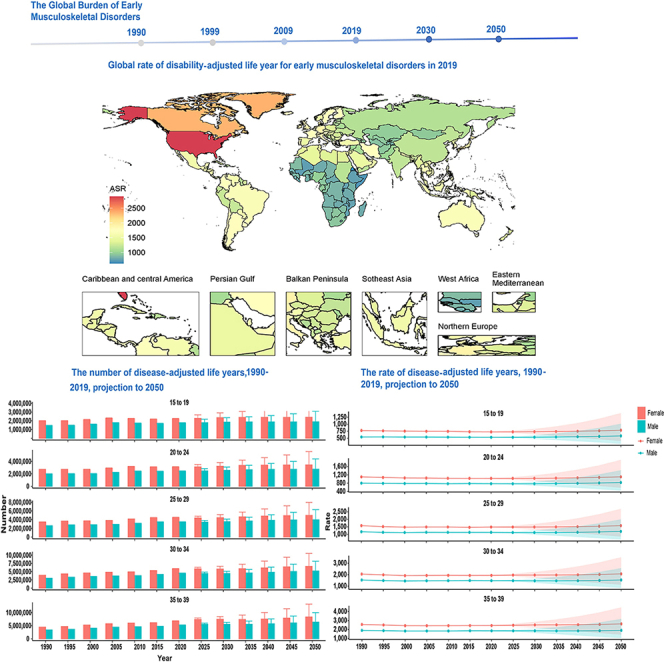

## Teaser

As adolescence and young adulthood have been largely overlooked in MSK health, the GBD 2019 MSK in Adolescents Collaborators estimate the burden, trends, forecasts, and disparities of early MSK disorders. They found an increasing burden of and cross-country inequalities in early MSK disorders, which might continue to increase by 2050.

## Introduction

Musculoskeletal (MSK) diseases encompass a wide range of conditions affecting the locomotor system, including joints, bones, tendons, muscles, ligaments, and the vertebral column. These conditions account for a significant global volume of years lived with disability (YLDs) and pose substantial threats to healthy aging by restricting physical and mental capacities and functional ability.^1^^,^^2^^,^^3^ Among these diseases, low back pain (LBP) and neck pain (NP) exert an enormous personal and socioeconomic burden on society, yet they receive only a fraction of the resources and attention dedicated to them.^4^^,^^5^ In addition, rheumatic and MSK disorders have a negative impact on the quality of life and contribute significantly to the overall burden of disability.^6^ Although pharmacological treatments exist for rheumatoid arthritis (RA) and gout, effectively reducing disease activity and improving disability, osteoarthritis (OA) lacks such efficacious treatment options.^7^^,^^8^^,^^9^ These concerning trends in major risk factors for MSK diseases underscore the importance of prevention during adolescence and young adulthood.^10^

Adolescence and young adulthood, spanning ages 15 to 39 years, represent a critical period during which risk factors accumulate. Without intervention, this can lead to a surge in disease burden during old age, especially considering that global life expectancy at birth increased from 67.2 to 73.5 years between 1990 and 2019.^11^ Furthermore, while there have been some improvements in ill health for the population aged ≥70, the rate of accumulating disease burden in adolescents and young adults is slower compared to that of those aged ≥70.^12^ Therefore, it is of utmost urgency to implement preventive measures and effective treatments in adolescents and young adults to improve the outcomes of MSK conditions, as older individuals place great value on maintaining independence and dignity.^13^

The prevalence and long-standing disparities of MSK diseases in adolescents and adults are fueled by inequities deeply rooted within society, as the foundation of MSK health is laid early in life. On one hand, the overall Healthcare Access and Quality Index ranges from 83.4 in high-sociodemographic-index (SDI) countries to 30.7 in low-SDI countries, and country-level universal health coverage effective coverage spans from 95 or higher in Japan and Iceland to lower than 25 in Somalia and the Central African Republic.^14^^,^^15^ In addition, there is a 10-fold difference in the global density of physicians between the highest-SDI countries and the lowest-SDI countries and a staggering 293.7 times difference in resources devoted to health between high-income and low-income countries.^16^^,^^17^

Despite concerning statistics about early MSK disease and the aging global population, little is known about how the disease burden, risk factors, and inequality have changed among individuals ages 15 to 39 years on a global, regional, and national scale. Therefore, this study aims to address the following questions: How did the disease burden, risk factors, and inequality change from 1990 to 2019? How will the disease burden change from 2020 to 2050? Are there sociodemographic-development-level-related inequalities in early MSK disease across countries?

## Results

### Global disease burden of early MSK disorders from 1990 to 2019 and projection from 2020 to 2050

**Figure 1:**
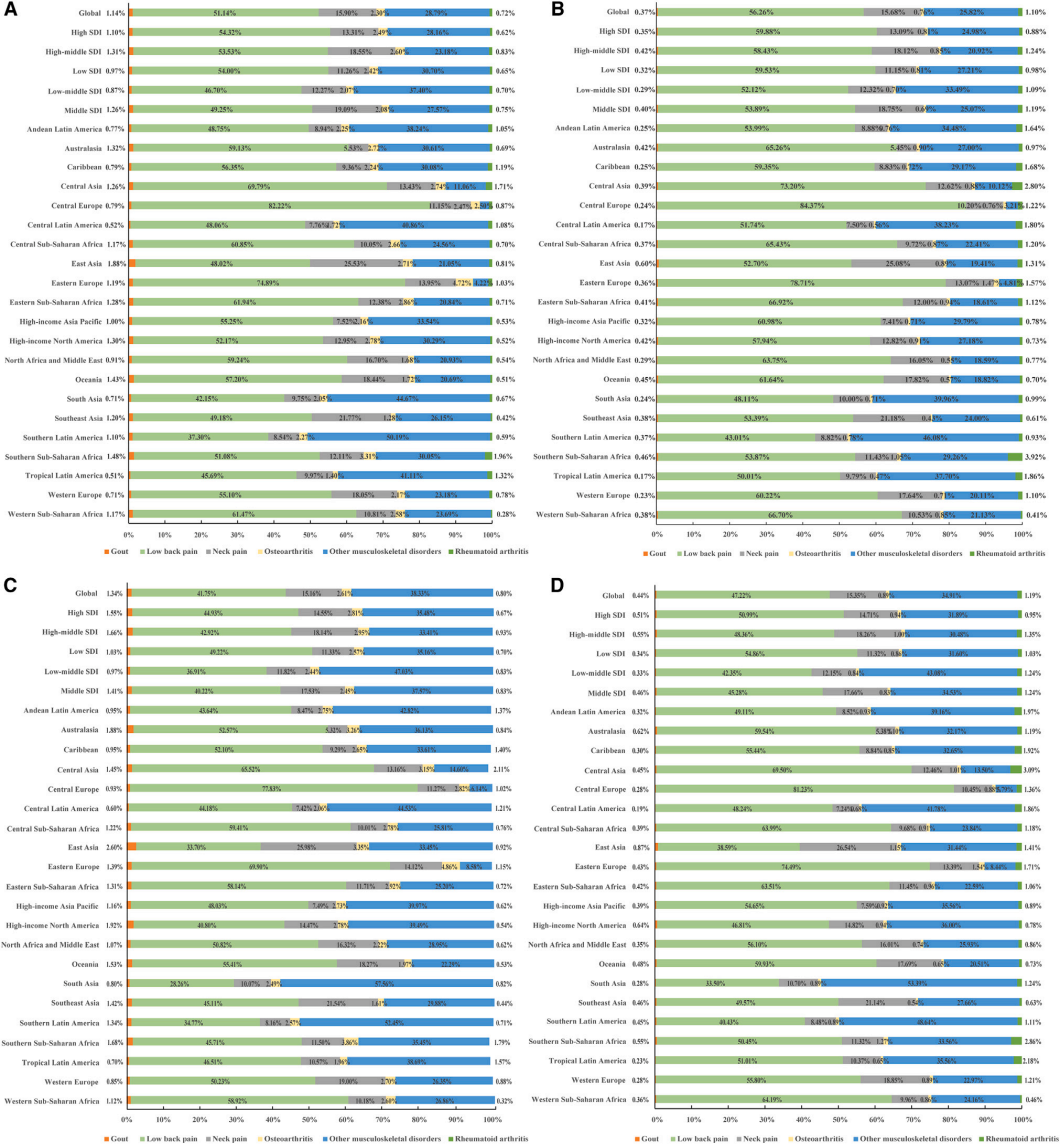
Contribution of prevalence and disability-adjusted life years from six individual to overall musculoskeletal (MSK) diseases, both sexes, globally and by region, 1990 and 2019 (A) Contribution of prevalence from six individual to overall MSK diseases, both sexes, globally and by region, 1990. (B) Contribution of disability-adjusted life years from six individual to overall MSK diseases, both sexes, globally and by region, 1990. (C) Contribution of prevalence from six individual to overall MSK diseases, both sexes, globally and by region, 2019. (D) Contribution of disability-adjusted life years from six individual to overall MSK diseases, both sexes, globally and by region, 2019.

**Figure 2:**
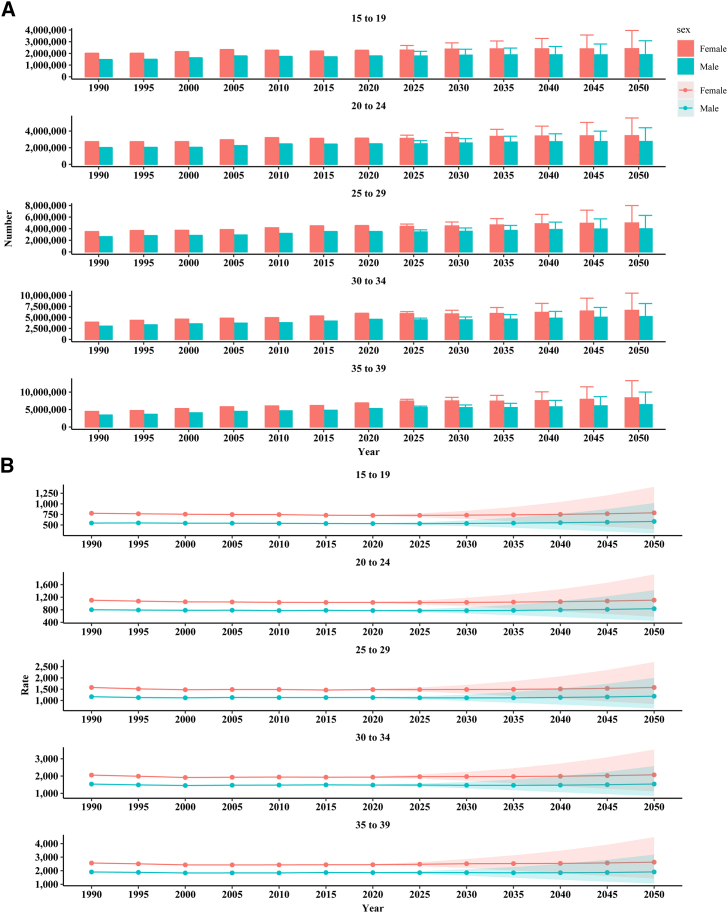
Projection of disease-adjusted life years by age from 2020 to 2050 (A) Projection of number of disease-adjusted life years by age from 2020 to 2050. (B) Projection of rate of disease-adjusted life years by age from 2020 to 2050.

From 1990 to 2019, the numbers of prevalence, disability-adjusted life years (DALYs), and YLDs for early MSK diseases increased with average annual percentage changes (AAPCs) of 1.15, 1.08, and 1.07, respectively, while the rates of prevalence, DALYs, and YLDs slightly rose, with AAPCs of 0.10, 0.02, and 0.02, respectively (Data S1; Tables S1 and S2). We observed an increased contribution of other MSK disorders (OMSKDs) but a decreased contribution of LBP to overall MSK diseases (Figure 1). Among the six MSK diseases, the numbers of prevalence and DALYs showed an upward trend from 1990 to 2019. However, the prevalence and DALY rates obviously declined for LBP, slightly decreased for NP, and notably increased for OMSKDs. Meanwhile, RA, OA, and gout remained relatively stable during this period (Figure S1). Except for gout, prevalence, DALYs, and YLDs were higher in females than in males for the other five MSK disorders (Data S1; Tables S1 and S2; Figure S2). The age-specific rates of DALYs and YLDs for overall MSK exhibited an upward trend in males, while a stagnant trend was observed in females (Data S1; Tables S1 and S2). Projections from 2020 to 2050 indicate that the age-specific number and rate of DALYs will universally continue to increase for both males and females across five age groups (Figure 2; Data S1; Tables S3 and S4).

### Global disease burden of early MSK disorders from 1990 to 2019 according to age group and SDI

Across five age groups, the rates of RA, NP, LBP, gout, and OMSKD prevalence as well as DALYs and YLDs sharply rose from ages 15–19 to 35–39. The prevalence rate of OA was negligible before age 30 but increased notably in the 30–34 group, rising at least 6-fold in the 35–39 group. More importantly, the MSK prevalence, DALYs, and YLDs increased faster from the 15–19 to the 35–39 age group, indicated by higher AAPC values. Generally, the prevalence rates, DALYs, and YLDs for gout, RA, OA, and OMSKDs showed an increasing trend across all age groups, but a decreasing trend was observed for LBP and NP (Data S1; Tables S5–S7). From 1990 to 2019, the numbers of prevalence and DALYs and YLDs for the six MSK diseases had the highest level of AAPC in low-SDI regions, except for the number of DALYs for overall MSK in the low- to middle-SDI region (Data S1; Tables S8–S10). At the same time, RA, OA, gout, and OMSKDs showed an upward trend across all five SDI regions. However, the prevalence rate of LBP decreased in all SDI regions and that of NP in the low- to middle-SDI region (Data S1; Table S8). Similar trends were observed in terms of DALY and YLD rates (Data S1; Tables S9 and S10).

### Global disease burden of early MSK disorders from 1990 to 2019 according to GBD region

**Figure 3:**
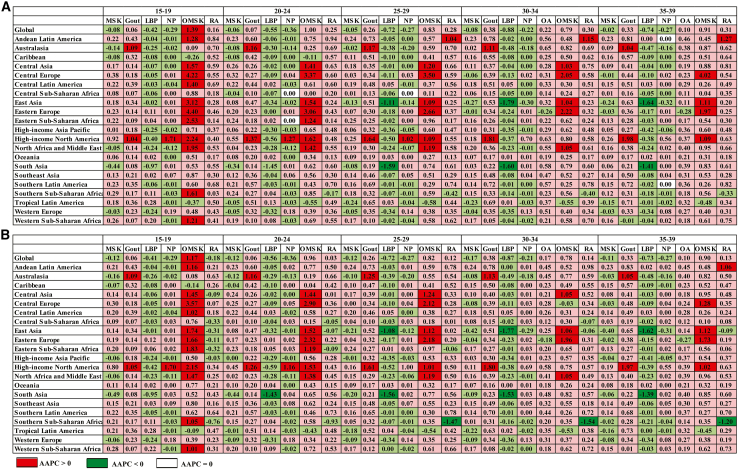
Average annual percentage change in rates of prevalence and disability-adjusted life years on musculoskeletal disorders among adolescents and young adults from 1990 to 2019, globally and regionally (A) Average annual percentage change in rate of prevalence of musculoskeletal disorders among adolescents and young adults from 1990 to 2019, globally and regionally. (B) Average annual percentage change in rate of disability-adjusted life years due to musculoskeletal disorders among adolescents and young adults from 1990 to 2019, globally and regionally. MSK, musculoskeletal disorders; LBP, low back pain; NP, neck pain; OA, osteoarthritis; OMSK, other musculoskeletal disorders; RA, rheumatoid arthritis.

The prevalence and DALY rates of overall MSK showed an upward trend in more than half of the Global Burden of Disease (GBD) study regions. A worsening trend (AAPC > 0) in prevalence and DALY rates of RA, OA, gout, and OMSKDs was observed in nearly all GBD regions of the world, while an improving trend (AAPC < 0) was seen in the prevalence and DALY rate of LBP from most GBD regions and NP from over half of the GBD regions (Figure 3). The most significant upward trend in the number of prevalent cases for overall MSK, LBP, and RA was observed in Western Sub-Saharan Africa; for NP in Central Sub-Saharan Africa; and for OMSKDs, OA, and gout in North Africa and the Middle East. Similar trends were found in the numbers of DALYs and YLDs, except for OMSKDs in Eastern Sub-Saharan Africa. The highest levels of AAPC for prevalence, DALY, and YLD rates for overall MSK disease, gout, LBP, NP, OA, OMSKDs, and RA were observed in North Africa and the Middle East, high-income North America, Tropical Latin America, high-income North America, North Africa and the Middle East, Central Europe, and Andean Latin America, respectively (Data S1; Tables S11–S13).

### Global disease burden of early MSK disorders from 1990 to 2019 by country and territory

Among 204 countries, in 2019, China, India, and the USA ranked as the top three in terms of prevalence and numbers of DALYs and YLDs for overall and six MSK disorders. The highest AAPC in prevalence and numbers of DALYs and YLDs were found in Qatar for overall MSK, gout, LBP, NP, OMSKD, and RA, while the highest AAPC in prevalence and numbers of DALYs and YLDs for OA were in the United Arab Emirates. However, the rates of prevalence, DALYs, and YLDs for overall MSK in the USA ranked first in 2019, with the highest AAPC in Lebanon. The highest rates of prevalence, DALYs, and YLDs for gout, LBP, NP, OA, OMSKD, and RA in 2019 were found in the USA, the USA, the USA, the USA, Canada, and Uzbekistan, with the highest AAPC in the Maldives, Vietnam (except the highest rate of prevalence for LBP in Canada), the Maldives, the Maldives, Eritrea, and Serbia, respectively (Data S1; Tables S14–S16; Figures S3–S6).

### Global disease burden of early MSK disorders attributable to risk factors from 1990 to 2019

From 1990 to 2019, the disease burden of LBP attributable to smoking decreased rapidly in the numbers of DALYs and YLDs, but there was an increasing trend in the disease burden of gout, LBP, and OA, attributable to high BMI and, in gout, to kidney disfunction (KD). The contribution of smoking to LBP and RA and of occupational ergonomic factors (OEFs) to LBP gradually decreased in terms of DALY and YLD rates, but the contribution of high BMI to gout, LBP, and OA and KD to gout quickly increased (Data S1; Table S17).

### The association between age-specific rate, sociodemographic index, and average annual percentage change

**Figure 4:**
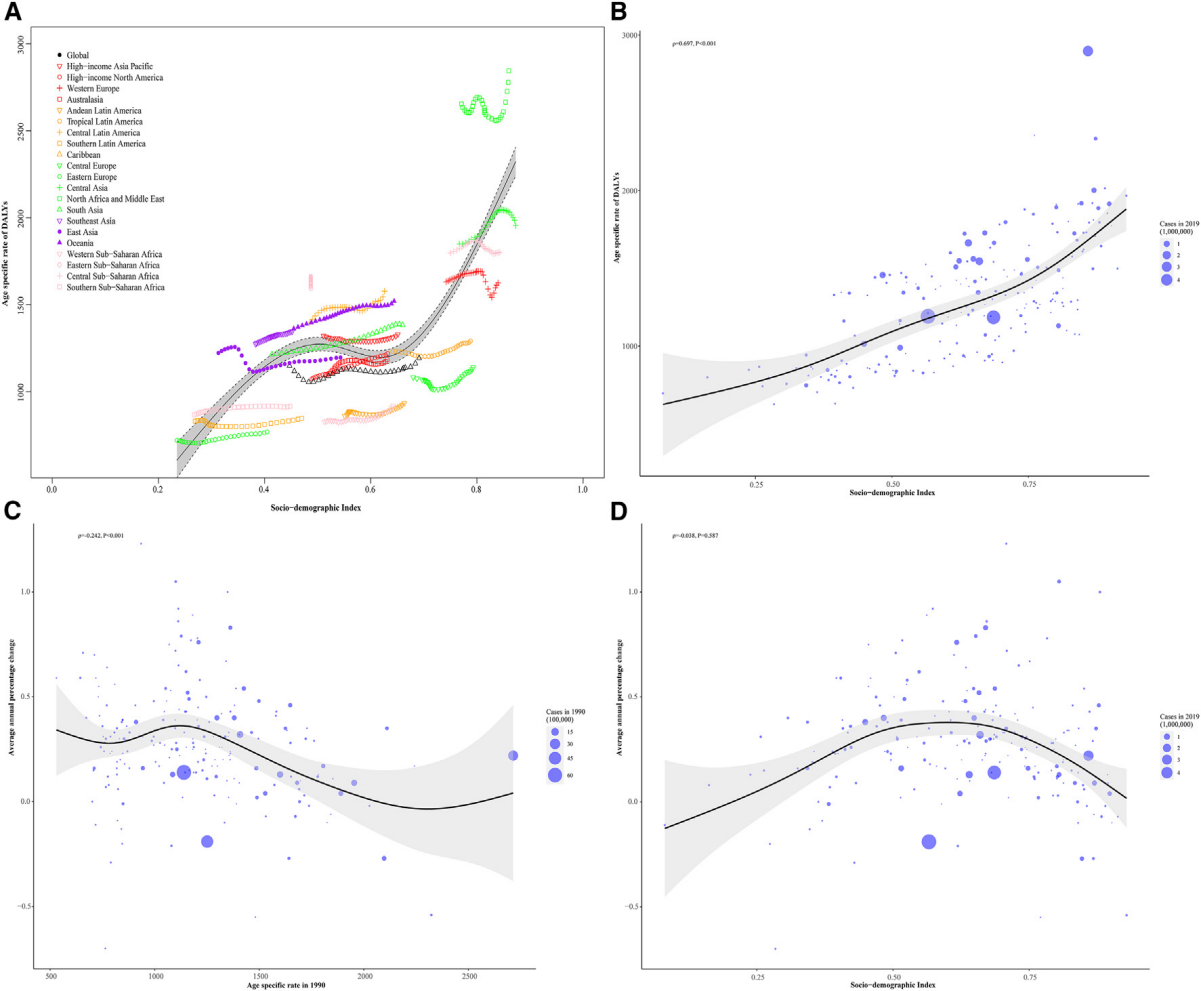
The association between age-specific rate of disease-adjusted life years and sociodemographic index for overall musculoskeletal disorders across GBD regions and countries and territories in 2019 (A) The association between age-specific rate of disease-adjusted life years and sociodemographic index for overall musculoskeletal disorders across 21 GBD regions in 2019. Black line represents expected values based on sociodemographic index and disease rates across 21 Global Burden of Disease regions; each point shows the observed age-specific rate of disease-adjusted life years for the specified Global Burden of Disease region in 2019. (B) The association between age-specific rate of disease-adjusted life years and sociodemographic index for overall musculoskeletal disorders across 204 countries and territories in 2019. Black line represents expected values based on sociodemographic index and disease across 204 countries and territories; each point shows the observed age-standardized rate of incidence for the specified country in 2019. (C) The correlation between average annual percentage change and age-specific rate of disease-adjusted life years attributable to overall musculoskeletal disorders in 1990 across 204 countries and territories. The size of the circle increases with the number of disease-adjusted life years. The ρ indices and *p* values were derived from Pearson correlation analysis. (D) The correlation between average annual percentage change and sociodemographic index attributable to overall musculoskeletal disorders in 2019 across 204 countries and territories. The size of the circle increases with the number of disease-adjusted life years. The ρ indices and *p* values were derived from Pearson correlation analysis.

There was a positive correlation between the SDI level and the age-specific rate of DALYs in 2019 across 21 GBD regions (r = 0.660, *p* < 0.001) and 204 countries and territories (r = 0.697, *p* < 0.001) (Figures 4A and 4B). Similar positive correlations were observed for gout, LBP, NP, OA, OMSKD, and RA (Data S1; Figures S7 and S8). The AAPC level was negatively correlated with the age-specific rate of DALYs in 1990 (r = −0.242, *p* < 0.001) (Figure 4C). Similar negative correlations were shown in LBP, OA, OMSKD, and RA but not in NP and gout (Data S1; Figure S9). By contrast, a positive correlation between AAPC level and SDI value in 2019 was observed when SDI was limited to below 0.50, but it gradually disappeared when SDI was above 0.50 (r = −0.038, *p* = 0.587) (Figure 4D). Similarly, no significant correlation was identified for the six MSK diseases (Data S1; Figure S10).

### The slope index of inequality and relative concentration index in 1990 and 2019

**Figure 5:**
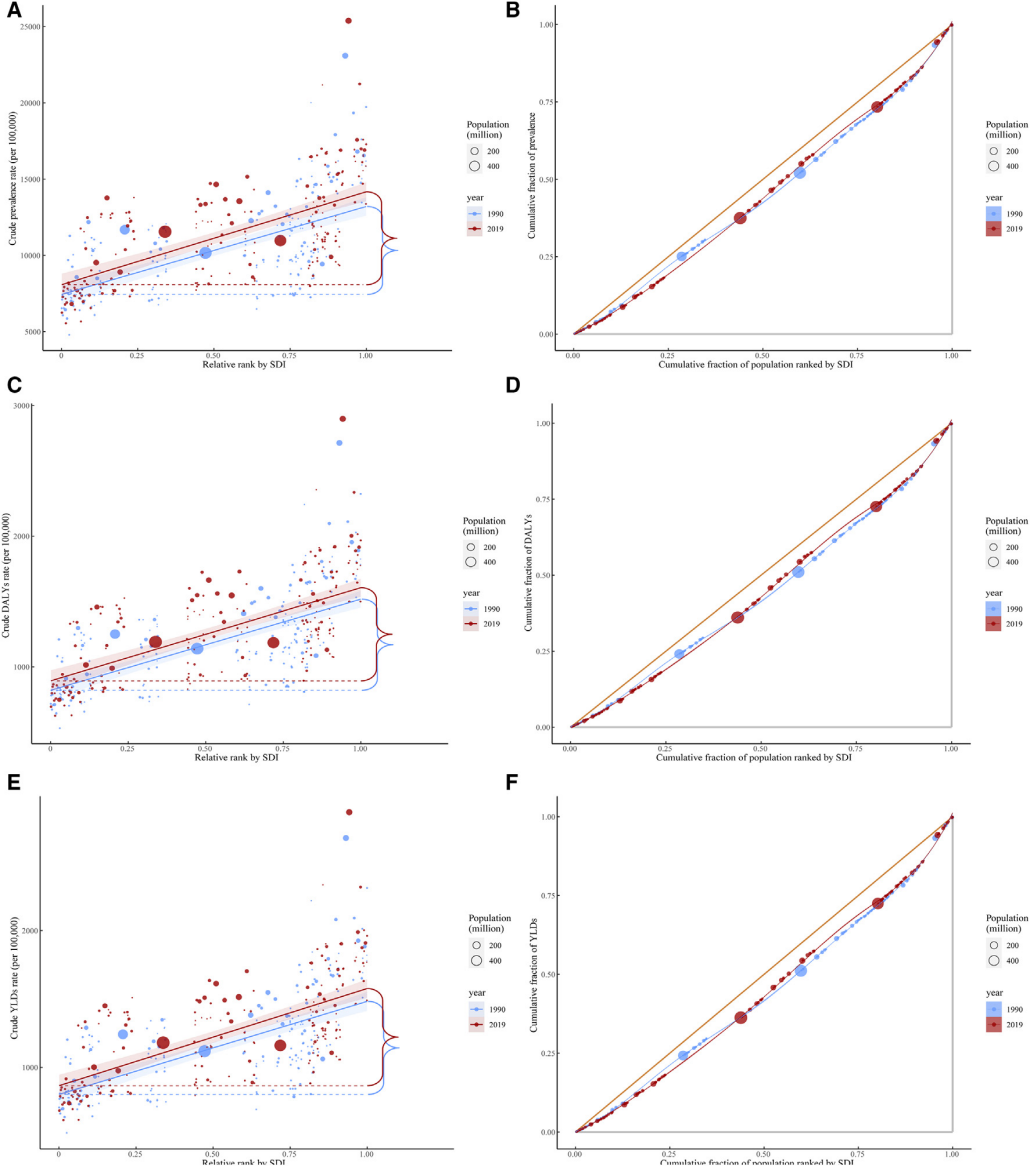
Health inequality regression curves and concentration curves for overall musculoskeletal disorders in terms of prevalence, disease-adjusted life years, and years lived with disability (A) Health inequality regression curves of prevalence for overall musculoskeletal disorders. (B) Concentration curves of prevalence for overall musculoskeletal disorders. (C) Health inequality regression curves of disease-adjusted life years for overall musculoskeletal disorders. (D) Concentration curves of disease-adjusted life years for overall musculoskeletal disorders. (E) Health inequality regression curves of years lived with disability for overall musculoskeletal disorders. (F) Concentration curves of years lived with disability for overall musculoskeletal disorders.

As indicated by the inequality slope index, the great gap in the prevalence, DALY, and YLD rates for overall MSK disorders between countries with the highest and the lowest SDI increased from 5,763.61, 697.43, and 680.59 in 1990 to 6,096.30, 713.23, and 710.72 in 2019, showing that countries with higher SDI bore disproportionately higher burdens. By contrast, the relative gradient inequality, as measured by the relative concentration index, was 10.10, 11.60, and 11.50 in 1990 and 11.30, 11.40, and 11.40 in 2019, showing a roughly proportionate concentration of the burden between the poor and the rich populations (Table 1; Figure 5). From 1990 to 2019, both the slope index of inequality and the relative concentration index increased for gout, NP, OA, and OMSKD, but there was a decreased relative concentration index and increased slope index of inequality for both LBP and RA (Table 1; Data S1; Figures S11–S13).

**Table 1:** Sociodemographic-index-related inequalities in prevalence, disease-adjusted life years, and years lived with disability for musculoskeletal diseases

Disease	Year	Inequality metrics	Prevalence (95% CI)	Disease-adjusted life years (95% CI)	Years lived with disability (95% CI)
Musculoskeletal disorders	1990	relative concentration index	10.10 (9.70, 10.50)	11.60 (10.50, 12.70)	11.50 (10.40, 12.60)
Musculoskeletal disorders	2019	relative concentration index	10.30 (10.00, 10.70)	11.40 (10.40, 12.30)	11.40 (10.40, 12.40)
Musculoskeletal disorders	1990	slope index of inequality	5,763.61 (4,791.05, 6,736.17)	697.43 (588.03, 806.83)	680.59 (570.86, 790.31)
Musculoskeletal disorders	2019	slope index of inequality	6,096.30 (5,103.93, 7,088.67)	713.23 (598.74, 827.73)	710.72 (596.07, 825.37)
Gout	1990	relative concentration index	11.40 (9.90, 13.00)	11.30 (9.10, 13.40)	11.30 (9.10, 13.40)
Gout	2019	relative concentration index	23.90 (21.10, 26.80)	23.70 (19.80, 27.60)	23.70 (19.80, 27.60)
Gout	1990	slope index of inequality	36.31 (20.86, 51.77)	1.26 (0.74, 1.78)	1.25 (0.73, 1.78)
Gout	2019	slope index of inequality	62.73 (46.10, 79.35)	2.12 (1.55, 2.69)	2.12 (1.55, 2.69)
Low back pain	1990	relative concentration index	14.60 (13.80, 15.30)	14.70 (13.20, 16.20)	14.70 (13.20, 16.20)
Low back pain	2019	relative concentration index	12.60 (12.10, 13.10)	12.80 (11.80, 13.70)	12.70 (11.80, 13.70)
Low back pain	1990	slope index of inequality	3,462.17 (2,935.73, 3,988.61)	405.91 (345.92, 465.89)	405.34 (344.97, 465.70)
Low back pain	2019	slope index of inequality	3,383.12 (2,880.34, 3,885.91)	394.35 (335.84, 452.85)	393.57 (335.65, 451.48)
Neck pain	1990	relative concentration index	11.00 (9.40, 12.70)	11.00 (8.70, 13.30)	11.00 (8.70, 13.30)
Neck pain	2019	relative concentration index	20.10 (17.50, 22.70)	20.10 (16.70, 23.50)	20.20 (16.70, 23.60)
Neck pain	1990	slope index of inequality	1,081.29 (761.91, 1,400.68)	111.83 (79.08, 144.57)	111.68 (78.94, 144.42)
Neck pain	2019	slope index of inequality	1,230.66 (898.94, 1,562.38)	126.23 (92.13, 160.33)	126.42 (92.44, 160.40)
Osteoarthritis	1990	relative concentration index	13.90 (12.80, 15.00)	13.50 (10.10, 17.00)	13.50 (10.10, 17.00)
Osteoarthritis	2019	relative concentration index	15.50 (14.50, 16.60)	15.30 (11.90, 18.80)	15.30 (11.90, 18.80)
Osteoarthritis	1990	slope index of inequality	138.04 (111.31, 164.78)	4.71 (3.83, 5.60)	4.71 (3.82, 5.59)
Osteoarthritis	2019	slope index of inequality	214.28 (184.22, 244.33)	7.27 (6.27, 8.27)	7.27 (6.27, 8.27)
Rheumatoid arthritis	1990	relative concentration index	12.60 (11.90, 13.20)	12.70 (11.40, 14.00)	12.30 (10.90, 13.70)
Rheumatoid arthritis	2019	relative concentration index	10.80 (10.20, 11.30)	10.90 (9.80, 11.90)	10.70 (9.60, 11.70)
Rheumatoid arthritis	1990	slope index of inequality	78.45 (66.81, 90.09)	11.18 (9.28, 13.08)	10.82 (9.22, 12.42)
Rheumatoid arthritis	2019	slope index of inequality	87.55 (71.81, 103.29)	12.01 (9.65, 14.37)	12.08 (9.93, 14.23)
Other musculoskeletal disorders	1990	relative concentration index	4.00 (3.70, 4.40)	5.00 (4.40, 5.60)	4.20 (3.60, 4.80)
Other musculoskeletal disorders	2019	relative concentration index	5.10 (4.70, 5.40)	5.30 (4.70, 6.00)	5.20 (4.50, 5.90)
Other musculoskeletal disorders	1990	slope index of inequality	2,002.96 (1,614.03, 2,391.88)	188.77 (148.68, 228.86)	178.81 (145.47, 212.15)
Other musculoskeletal disorders	2019	slope index of inequality	2,335.66 (1,762.21, 2,909.12)	207.03 (147.24, 266.81)	207.68 (156.52, 258.85)

## Discussion

Early MSK disorders are increasingly prevalent and substantially affect the disease burden. Our study revealed a clear upward trend in the burden of early MSK disease from 1990 to 2019. These findings call for urgent global actions to address early MSK disease. There is also a sense of urgency in tackling the disparate burden of early MSK disease, given the paradoxical trends of increasing rates for gout, OA, RA, and OMSKDs but decreasing burden from LBP and NP. In addition, there was an increasing burden in countries with middle, middle-high, and high SDI, and females were found to be more susceptible to early MSK disease. These findings indicate the urgent need for measures to control the spread in developed countries and among female adolescents and young adults. Furthermore, we observed an increasing contribution of high BMI to gout, LBP, and OA and the contribution of KD to gout. Therefore, weight control and management of KD are essential in reducing the burden of early MSK disease.

Looking ahead to the next 30 years, the global burden of early MSK disease will continue to grow. First, the projected increase in the numbers and rates of people living with early MSK disease can be attributed to population growth and early onset of MSK disease. With increasing life expectancy, the global burden of MSK diseases is bound to rise further if these early MSK diseases are not effectively addressed. Second, low-SDI regions have strong potential to increase the burden without well-established prevention and treatment systems. Third, estimates show that high BMI and KD are key modifiable factors in mitigating the increasing burden of MSK disease.^18^ Fourth, early MSK disease burden will continue to increase in the coming years, as available interventions can only taper this substantial disease burden; these interventions include biologic agents for RA, urate-lowering therapy for gout, and cognitive functional therapy for LBP. Fifth, the projected increase in DALYs will disproportionately affect females more than males. These projections motivate policymakers to implement a more targeted sex- and culturally specific approach with an emphasis on risk stratification and interventions focused on tackling the root drivers of MSK disease in ever-changing populations. Accelerated changes in or reversal of recent trends would be expected to increase or decrease future MSK disease burden, as the projections, based on existing data, assume that these driving factors will continue to spread with increasing urbanization and development.

Our findings imply that countries with higher SDI share an overwhelming burden of early MSK disease. Three reasons explain why individuals from higher SDI countries, who have more access to better health and medical services, shoulder a higher early MSK disease burden. First, MSK diseases are incurable conditions characterized by a chronic long-term disease course and recurrent flares, despite occurring in developed countries, leading to a huge demand for medical care. Second, BMI is increasing considerably faster than smoking, OEFs, and KD.^10^ There is a long way for us to go in controlling BMI at the national level via modifying the nexus of physical inactivity, diet quality, and excess energy intake. Third, our discovery of significantly increasing socioeconomic inequalities in early MSK disease burden between countries over time is unacceptable and represents a significant gap in current disease prevention and management efforts. On the one hand, countries with vast territories and large populations will have greater challenges in fighting MSK disease, as developing and strengthening control programs are much more complex and expensive to implement. On the other hand, the unique growing rural-urban and within-rural disparities often go unnoticed, despite many rural communities experiencing population decline.^19^ Notably, there was a modest decrease from 1990 to 2019 in the relative concentration index and slope index of inequality for LBP, which might be driven by changes in manual labor or increased recovery.

Patients with MSK disease during adolescence and young adulthood represent a population with a substantial number of potential life years saved but are at a higher risk of developing long-term morbidity or dying prematurely. There are exclusive opportunities available for individuals ages 15–19 to alleviate the disease burden associated with RA, NP, LBP, gout, and OMSKD and for those ages 30–34 to address OA-related burden. Public health approaches, such as tobacco and sugar-sweetened beverage taxes, family leave, and early childhood education, can result in population-wide improvements in MSK health starting early in life. At the system level, it is crucial to fill the gap of medical care transitioning from pediatric to adult care by increasing the treatment and control rates and engaging adolescents and young adults in the health care system. At the individual level, approaches tailored to the needs and preferences of adolescents and young adults, such as comfort with technology and optimal frequency of screening for risk determinants, are important. In the clinical setting, continued research is needed to ascertain effective lifestyle and pharmacological strategies to control non-optimal risk determinant levels and preserve optimal risk factor levels.

Further high-quality epidemiological studies should change the focus from sex-, geographical-, and socioeconomic-specific factors to the effects of screening, detection, and prevention programs on MSK disease burden and inequality. The utility of digital health applications in the management of MSK disease needed to be disease and instrument specific, as a positive effect of a smart system of disease management in RA but a neutral effect of an artificial-intelligence-based self-management app in LBP and NP were observed.^20^^,^^21^ Whether opioid analgesics could reduce the disease burden of LBP and NP needs to be further examined, as a new randomized trial did not lend support to usage of opioids when other pharmacological treatments are contraindicated or have not worked.^22^ It is of great importance for us to understand the effects of vaccination in preventing post-COVID inflammatory arthritis and the mechanisms implicated in the development of RA after COVID-19.^23^ The incidence of RA especially increased during the first year following the diagnosis of COVID-19, and the age group hazard ratios increased until the age group of 51–60 years and then decreased in the age group of 61 years or older compared to those ages 18–30 years.^23^ Future translational studies are warranted to explore the effects of novel drugs on MSK diseases, such as PD-1 agonist (peresolimab) and antibody-drug conjugate (composed of anti-TNF monoclonal antibody linked to a glucocorticoid receptor modulator) for RA.^24^^,^^25^ More smart designed studies targeting specific subtypes of OA are needed to investigate the role of inflammation in OA and potential structure-modifying effects of anti-inflammatory drugs for the treatment of OA.^26^^,^^27^ The importance of lifestyle modifications and optimization of comorbidities cannot be degraded, even though the enthusiasm for discovering novel therapeutics and their benefits on MSK health is ever increasing.

### Limitations of the study

Our study has limitations in addition to the inherent weaknesses of GBD methodology, although GBD employs a combination of methodological and statistical approaches to manage the quality of the primary study and heterogeneity of original data (https://www.healthdata.org/research-analysis/about-gbd). First, our predicted results did not allow observed time trends to affect the forecasted disease burden in the future, as it might induce spurious trends caused by changes in diagnostic procedures over time. Our projections of disease burden are based solely on expected trends in prevalence as well as trends in population aging and growth. Second, we were unable to perform subgroup analyses between urban and rural areas, which remain an important area for future research. Third, the absence of province-level and urban-rural stratification in the results of GBD 2019 could potentially introduce bias into the analysis of disparities and hinder the formulation of targeted health policies. Fourth, the study failed to assess the effects of diverse health systems across various nations or regions due to significant disparities in health advancements between nations with similar SDIs. Fifth, the study did not provide a separate assessment of each disease included in OMSKDs beyond the five major MSK disorders, which may result in an underestimation of the disease burden of these additional MSK disorders. Particularly, the disease burden attributable to osteoporosis and low bone mineral density might be underestimated due to several factors: (1) limited access to screening programs in low-SDI regions; (2) time lag between osteoporotic fractures and mortality; (3) higher costs and technical complexities associated with screening and testing equipment compared to other major chronic conditions like hypertension; (4) varied parameters of dual-energy X-ray absorptiometry devices and different diagnostic standards for osteoporosis from organizations such as the WHO, International Society for Clinical Densitometry, and National Osteoporosis Foundation; (5) exclusion of additional osteoporotic fracture sites, which may not be the primary factor in fatality; and (6) low sensitivity of bone mineral density in diagnosing fracture risk.^28^^,^^29^^,^^30^^,^^31^ Sixth, the Joinpoint analysis of modeled data might lead to an underestimation of the uncertainty in AAPC trends, as uncertainty in GBD methodology was not considered in the regression analysis. Seventh, many important MSK diseases, such as systemic lupus erythematosus and spondylarthritis, were mapped to OMSKDs based on the ICD10 mapping methodology. Finally, cross-country social inequalities analysis is cross-national, which may introduce bias due to a lack of knowledge of disparities that exist between districts within countries.

## STAR★Methods

### Key resources table

**Table:** 

REAGENT or RESOURCE	SOURCE	IDENTIFIER
**Software and algorithms**		

R 4.3.0	R	http://www.r-project.org/
Joinpoint	Joinpoint	https://surveillance.cancer.gov/joinpoint/
2019 Global Burden of Disease study	IHME	https://www.healthdata.org/; https://vizhub.healthdata.org/gbd-results/

### Resource availability

#### Lead contact

Further information and requests for resources should be directed to and will be fulfilled by the lead contact, Dr. Dongze Wu (dongze_wu@163.com).

#### Materials availability

This study did not generate new unique reagents.

#### Data and code availability

• All data reported in this paper are publicly available from the Institute of Health Metrics and Evaluation (http://www.healthdata.org/; http://ghdx.healthdata.org/gbd-results-tool).
• This paper does not report original code.

### Experimental model and study participant details

This study did not enroll study participants.

### Method details

#### Data sources

The Global Burden of Disease (GBD) 2019 project estimated the disease burden associated with 369 diseases and injuries for 204 countries and territories from 1990 to 2019.^3^^,^^10^ [3, 10]. The study estimated the prevalence, disability-adjusted life years (DALYs), years lived with disability (YLDs) of musculoskeletal (MSK) diseases, including rheumatoid arthritis (RA), osteoarthritis (OA), LBP, NP, gout, and other musculoskeletal disorders (OMSKD). Additionally, it examined their attributable risk factors, such as smoking, occupational ergonomic factors (OEF), high body-mass index (BMI), and kidney dysfunction (KD). Our study specifically focused on MSK disorders occurring between the ages of 15 and 39 years (defined as adolescent and young adult). The detailed methodology used to estimate the disease burden of MSKs is described in the accompanying GBD 2019 publications.^3^^,^^10^ The estimates and methods used in this study are publicly available from the Institute for Health Metrics and Evaluation website, including the GBD Compare tool (https://vizhub.healthdata.org/gbd-compare/) and the GBD Results Tool (http://ghdx.healthdata.org/gbd-results-tool).

#### Case definition

##### Musculoskeletal disorders

This category encompasses mortality and predominantly disability arising from rheumatoid arthritis, osteoarthritis, low back pain, neck pain, gout, and a broad residual group of various other musculoskeletal disorders.

##### Low back pain (LBP)

LBP is characterized by pain in the lower part of the back, with or without referred pain into one or both lower limbs, lasting for a minimum of one day. The term "low back" refers to the region on the back's posterior aspect, extending from the lower margin of the twelfth ribs to the lower gluteal folds.

The ICD-10 codes for low back pain are M54.3, M54.4, and M54.5, while the corresponding ICD-9 code is 724.

##### Neck pain (NP)

Neck pain (NP) is characterized as pain in the neck, with or without referred pain into the upper limb(s), lasting for a minimum of one day.

The ICD-10 code for neck pain is M54.2, and the corresponding ICD-9 code is 723.1.

##### Gout

Gout is a rheumatic condition characterized by the accumulation of monosodium urate (MSU) crystals in the synovial fluid of joints and other tissues, leading to inflammation. The formation of these crystals is a result of elevated urate levels in extracellular fluids. The Global Burden of Disease (GBD) adopts the primary gout case definition provided by the American College of Rheumatology, commonly known as the ARA 1977 survey criteria. This definition necessitates the presence of MSU crystals in joint fluid or a tophus confirmed to contain MSU crystals, along with a minimum of six out of 12 specified gout symptoms or findings. These include experiencing more than one episode of acute arthritis, the onset of maximum inflammation within a day, an attack of monoarticular arthritis, observation of joint erythema, pain or swelling in the first metatarsophalangeal (MTP) joint, unilateral attacks involving the first MTP joint or tarsal joint, suspected tophus, hyperuricemia, asymmetrical swelling within a joint visible on X-ray, and negative culture of joint fluid for microorganisms during an inflammatory joint episode.

The ICD-10 code for gout is M10, and the ICD-9 code is 274.

##### Osteoarthritis (OA)

The definition used as a standard reference for OA involves the presence of symptomatic OA, which has been radiologically verified to be at Kellgren-Lawrence grade 2–4. In the GBD 2019 framework, two additional categories of OA were introduced. The first is OA affecting the hand, which meets the same reference criteria as any single hand joint type. The second category is OA occurring in joints other than those found in the hand, hip, knee, or spine, and it also adheres to the same reference criteria.

For symptomatic OA at grade 2, it is necessary to have one clearly defined osteophyte in the affected joint, along with experiencing pain for at least one month within the last 12 months. Grade 3–4 symptomatic OA requires the presence of osteophytes and narrowing of the joint space in the affected joint, with deformity present in grade 4. Similar to grade 2, pain must be experienced for at least one month out of the past 12 months.

##### Other musculoskeletal disorders

Other musculoskeletal disorders are a diverse residual group encompassing a broad spectrum of conditions affecting muscles, bones, and ligaments. These conditions are not encompassed within the five musculoskeletal diseases defined by the Global Burden of Disease (GBD): rheumatoid arthritis, osteoarthritis, low back and neck pain, and gout. Additionally, they are not classified as long-term sequelae resulting from injuries. A predominant number of fatalities attributed to other musculoskeletal disorders from autoimmune conditions like systemic lupus erythematosus and systemic sclerosis.

##### Rheumatoid arthritis (RA)

RA is a systemic autoimmune disorder characterized by joint pain, swelling, and deformities, often accompanied by systemic symptoms. While RA is acknowledged for its impact on internal organs alongside joints, these extra-articular effects are currently not quantified in the Global Burden of Disease (GBD).

The standard definition for RA in GBD is based on the 1987 criteria established by the American College of Rheumatology (ACR 1987). These criteria include seven diagnostic factors, of which at least four must be met for a diagnosis, and the first four must have been present for a minimum of six weeks: Morning stiffness; Arthritis affecting three or more joint areas; Arthritis affecting hand joints; Symmetric arthritis; Rheumatoid nodules; Serum rheumatoid factor; Radiographic changes.

For RA, the ICD-10 codes are M05, M06, and M08, while the ICD-9 codes range from 714.0 to 714.9.

#### ICD-10 and ICD-9 codes

**Table:** 

Cause	ICD10	ICD9
Musculoskeletal disorders	L93, M00–M02, M05–M06.9, M08.0–M08.8, M08, M10, M11–M13, M16, M17, M18, M19, M20–M25, M30–M35, M40–M43, M45–M46, M54.2, M54.3, M54.4, M54.5, M60–M63, M65–M68, M70–M73, M75–M79, M80–M85, M86, M87–M90, M91–M94, M95–M99	274, 710.0, 711, 712–713, 714–714.3, 714.8–714.9, 715, 716–719, 710.1–710.9, 737, 720–721, 723.1, 724, 725, 726–728, 729, 733.0–2, 730.1–730.3, 730.7–9, 731, 733.3–9, 732, 734–736, 738–739
Rheumatoid arthritis	M05–M06.9, M08.0–M08.8	714–714.3, 714.8–714.9
Osteoarthritis	M16, M17, M18, M19	715
Low back pain	M54.3, M54.4, M54.5	724
Neck pain	M54.2	723.1
Gout	M10	274
Other musculoskeletal disorders	L93, M00–M02, M08, M11–M13, M20–M25, M30–M35, M40–M43, M45–M46, M60–M63, M65–M68, M70–M73, M75–M79, M80–M85, M86, M87–M90, M91–M94, M95–M99	710.0, 711, 712–713, 716–719, 710.1–710.9, 737, 720–721, 725, 726–728, 729, 733.0–2, 730.1–730.3, 730.7–9, 731, 733.3–9, 732, 734–736, 738–739
Lupus erythematosus	L93	710.0
Infectious arthropathies	M00–M02	711
Inflammatory polyarthropathies	M08, M11–M13	712–713
Other joint disorders	M20–M25	716–719
Systemic connective tissue disorders	M30–M35	710.1–710.9
Deforming dorsopathies	M40–M43	737
Spondylopathies	M45–M46	720–721
Disorders of muscles	M60–M63	725
Disorders of synovium and tendon	M65–M68	726–728
Other soft tissue disorders	M70–M73, M75–M79	729
Disorders of bone density and structure	M80–M85	733.0–2
Osteomyelitis	M86	730.1–730.3, 730.7–9
Other osteopathies	M87–M90	731, 733.3–9
Chondropathies	M91–M94	732
Other disorders of the MSK system and connective tissue	M95–M99	734–736, 738–739

#### Definition of glossary

##### Cross-country inequality

The inequalities between countries based on national SDI level.

##### Years lived with disability (YLDs)

The calculation of YLDs involves multiplying the prevalence of MSK conditions by the disability weights assigned to each level of severity.

##### Disability-adjusted life years (DALYs)

Years of Live Lost (YLLs) for fatal causes were determined by calculating the difference between the observed deaths and the reference standard life expectancy at the age of death. The reference standard life expectancy data were derived from the GBD standard life table. DALYs, which serve as a comprehensive indicator of total health loss, were calculated by summing the YLLs and YLDs for each cause within the category of mental and substance use disorders.

##### Socio-demographic index

The sociodemographic index (SDI) is a composite index of socio-demographic development status strongly correlated with health outcomes. It represents the mean education level for those aged 15 years or older, the geometric mean of 0 to 1 indices of the total fertility rate in those under 25 years old, and lag-distributed income per capita.

#### Cross-country inequality analysis

The slope index and relative concentration index of inequality are used to quantify the distributive inequality of MSK disease burden across countries.^32^^,^^33^ The slope index of inequality is calculated by regressing the national rate of DALYs in the population aged 15–39 years on an SDI-associated relative position scale, defined by the midpoint of the cumulative class interval of the population ranked by gross domestic product per capita. The weighted regression model and a logarithmic transformation of the relative social position value were used to account for heteroskedasticity and non-linearity due to marginal utility. The concentration index is calculated by numerical integration of the area under the Lorenz concentration curve, which was fitted using the cumulative fraction of DALYs and cumulative relative distribution of the population ranked by SDI.

##### Relative concentration index

(RCI) demonstrates gradients among population subgroups on a relative scale. It indicates the degree to which an indicator is concentrated within subgroups that are either disadvantaged or advantaged. RCI is a relative measure of inequality that considers all demographic subgroups within a population. It is computed for ordered dimensions that have more than two subgroups, such as economic status. Subgroups are assigned weights based on their share of the total population.

##### Calculation

Dividing Absolute Concentration Index (ACI) by the designated average value (***μ***), followed by multiplying the resulting fraction by 100:

$$\text{RCI}=\frac{\text{ACI}}{\mu }*100$$

##### Interpretation

The RCI score falls within a range of −100 to +100, and it registers as zero when there is no inequality. When RCI is positive, it signifies that the indicator is more concentrated among the advantaged group, whereas negative RCI values indicate a concentration of the indicator among the disadvantaged. A larger absolute RCI value corresponds to a higher degree of inequality.

##### Slope index of inequality (SII)

SII quantifies the disparity in estimated indicator values between the most privileged and least privileged groups (or vice versa for negative indicators). This calculation incorporates all other subgroups, utilizing a suitable regression model. SII serves as an absolute gauge of inequality that considers all demographic subgroups, and its computation involves the proportional weighting of subgroups based on their population distribution.

##### Calculation

To derive the Slope Index of Inequality (SII), a weighted sample representing the entire population is organized in a ranked order, with the most disadvantaged subgroup assigned rank 0 and the most advantaged subgroup assigned rank 1. This ranking is weighted to reflect the proportional distribution of the population within each subgroup. The population within each subgroup is then analyzed in terms of its position within the cumulative population distribution and the midpoint of this range is determined. In accordance with the definition currently employed in Health Equity Assessment Toolkit (HEAT) plus software (https://www.who.int/data/inequality-monitor/assessment_toolkit), the indicator of interest is subsequently subjected to regression against this midpoint value using a generalized linear model with a logit link. The model calculates predicted indicator values for the two extremes (rank 1 and rank 0). In the case of favorable indicators, the SII value is computed as the difference between the estimated values at rank 1 (*v*1) and rank 0 (*v*0), covering the entire distribution.

SII=v1−v0

In the case of unfavorable indicators, the computation is reversed, and the SII value is determined as the disparity between the estimated values at rank 0 (*v*0) and rank 1 (*v*1), encompassing the entire distribution:

SII=v0−v1

##### Interpretation

When there's no inequality, the Social Inequality Index (SII) is at zero. Larger absolute values signify increased levels of inequality. In the case of positive values associated with positive indicators, it suggests that the indicator is more concentrated among the privileged, while negative values suggest a concentration among the underprivileged. Conversely, for negative indicators, positive values indicate a concentration among the disadvantaged, while negative values indicate a concentration among the privileged.

#### Projection analysis

The projection analysis is designed to implement Bayesian age-period-cohort models, with a specific emphasis on projections. Bayesian age-period-cohort models (BAPC) employ integrated nested Laplace approximations (INLA) to facilitate comprehensive Bayesian inference.^34^ It could produce age-specific, and age-standardized projected rates. In cases where the focus is on the predictive distribution, Poisson noise is automatically incorporated.

### Quantification and statistical analysis

The study compared the prevalence, DALYs, and YLDs between the sexes, causes (six MSK diseases), age groups (five-year intervals: 15–19, 20–24, 25–29, 30–34, 35–39 years), SDI (five categories), regions (21 GBD regions), and countries (204 countries and territories). The temporal trend was evaluated using a Join-point Regression Program (Version 4.8.0.1, Statistical Methodology and Applications Branch, Surveillance Research Program, National Cancer Institute), and the average annual percent change (AAPC) was calculated during 1990–2019, while the annual percent change (APC) was calculated during 1990–1999, 2000–2009, and 2010–2019, respectively. The BAPC model integrated nested Laplace approximations were used to project the DALYs of MSK disease from 2020 to 2050 via the R package BAPC and INLA. Cross-country inequality analysis and visualization were executed using the Health Equity Assessment Toolkit from WHO and R software (R-4.2.3).^32^ To investigate the factors influencing AAPCs, the association between AAPCs and ASRs (1990) and SDI (2019) was assessed at the national level using generalized linear model (GLM). A significance level of *p* < 0.05, at a two-tailed level, was used to determine statistical significance.

## Consortia

The members of GBD 2019 MSK in Adolescents Collaborators are Yingzhao Jin, Cui Guo, Mohammadreza Abbasian, Mitra Abbasifard, J. Haxby Abbott, Auwal Abdullahi, Aidin Abedi, Hassan Abidi, Hassan Abolhassani, Eman Abu-Gharbieh, Salahdein Aburuz, Ahmed Abu-Zaid, Isaac Yeboah Addo, Oyelola A. Adegboye, Abiola Victor Adepoju, Wirawan Adikusuma, Qorinah Estiningtyas Sakilah Adnani, Shahin Aghamiri, Danish Ahmad, Ayman Ahmed, Janardhana P. Aithala, Shiva Akhlaghi, Sreelatha Akkala, Tariq A. Alalwan, Mohammed Albashtawy, Hediyeh Alemi, Fadwa Alhalaiqa Naji Alhalaiqa, Endale Alemayehu Ali, Sami Almustanyir, Rajaa M. Al-Raddadi, Nelson J. Alvis-Zakzuk, Yaser Mohammed Al-Worafi, Hosam Alzahrani, Karem H. Alzoubi, Sohrab Amiri, Hubert Amu, Jimoh Amzat, David B. Anderson, Abhishek Anil, Benny Antony, Jalal Arabloo, Damelash Areda, Al Artaman, Anton A. Artamonov, Krishna K. Aryal, Mohammad Asghari-Jafarabadi, Tahira Ashraf, Seyyed Shamsadin Athari, Bantalem Tilaye Atinafu, Maha Moh’d Wahbi Atout, Sina Azadnajafabad, Hamed Azhdari Tehrani, Ahmed Y. Azzam, Alaa Badawi, Nayereh Baghcheghi, Ruhai Bai, Vali Baigi, Maciej Banach, Morteza Banakar, Biswajit Banik, Mainak Bardhan, Till Winfried Bärnighausen, Hiba Jawdat Barqawi, Amadou Barrow, Azadeh Bashiri, Kavita Batra, Mojtaba Bayani, Nebiyou Simegnew Bayileyegn, Ahmet Begde, Kebede A. Beyene, Akshaya Srikanth Bhagavathula, Pankaj Bhardwaj, Gurjit Kaur Bhatti, Jasvinder Singh Bhatti, Rajbir Bhatti, Ali Bijani, Veera R. Bitra, Javier Brazo-Sayavera, Rachelle Buchbinder, Katrin Burkart, Yasser Bustanji, Muhammad Hammad Butt, Luis Alberto Cámera, Felix Carvalho, Vijay Kumar Chattu, Akhilanand Chaurasia, Guangjin Chen, Haowei Chen, Lingxiao Chen, Steffan Wittrup McPhee Christensen, Dinh-Toi Chu, Isaac Sunday Chukwu, Josielli Comachio, Natália Cruz-Martins, Sarah Cuschieri, Sriharsha Dadana, Omid Dadras, Xiaochen Dai, Zhaoli Dai, Saswati Das, Mohsen Dashti, Ivan Delgado-Enciso, Biniyam Demisse, Edgar Denova-Gutiérrez, Belay Desye, Syed Masudur Rahman Dewan, Sameer Dhingra, Mengistie Diress, Thanh Chi Do, Thao Huynh Phuong Do, Khanh Duy Khanh Doan, Sulagna Dutta, Arkadiusz Marian Dziedzic, Hisham Atan Edinur, Michael Ekholuenetale, Muhammed Elhadi, Sharareh Eskandarieh, Francesco Esposito, Adeniyi Francis Fagbamigbe, Parisa Farokh, Ali Fatehizadeh, Alireza Feizkhah, Ginenus Fekadu, Nuno Ferreira, Getahun Fetensa, Florian Fischer, Behzad Foroutan, Masoumeh Foroutan Koudehi, Richard Charles Franklin, Takeshi Fukumoto, Aravind P. Gandhi, Balasankar Ganesan, Shuo-Yan Gau, Rupesh K. Gautam, Abadi Kahsu Gebre, Miglas W.W. Gebregergis, Bardiya Ghaderi Yazdi, Ali Gholami, Tiffany K. Gill, Pouya Goleij, Mansueto Gomes-Neto, Anmol Goyal, Simon Matthew Graham, Bin Guan, Bhawna Gupta, Indarchand Ratanlal Gupta, Sapna Gupta, Veer Bala Gupta, Vivek Kumar Gupta, Farrokh Habibzadeh, Wase Benti Hailu, Ramtin Hajibeygi, Rabih Halwani, Josep Maria Haro, Jan Hartvigsen, Ahmed I. Hasaballah, Johannes Haubold, Jeffrey J. Hebert, Mohamed I. Hegazy, Golnaz Heidari, Mohammad Heidari, Kamal Hezam, Yuta Hiraike, Hassan Hosseinzadeh, Mehdi Hosseinzadeh, Amir Human Hoveidaei, Chi-Jen Hsu, Md Nazmul Huda, Hong-Han Huynh, Bing-Fang Hwang, Segun Emmanuel Ibitoye, Adalia I. Ikiroma, Irena M. Ilic, Milena D. Ilic, Arad Iranmehr, Sheikh Mohammed Shariful Islam, Nahlah Elkudssiah Ismail, Hiroyasu Iso, Masao Iwagami, Assefa N. Iyasu, Louis Jacob, Abdollah Jafarzadeh, Kasra Jahankhani, Nityanand Jain, Ammar Abdulrahman Jairoun, Balamurugan Janakiraman, Umesh Jayarajah, Shubha Jayaram, Jayakumar Jeganathan, Mohammad Jokar, Jost B. Jonas, Tamas Joo, Nitin Joseph, Charity Ehimwenma Joshua, Gebisa Guyasa Kabito, Vineet Kumar Kamal, Himal Kandel, Rami S. Kantar, Jafar Karami, Ibraheem M. Karaye, Arman Karimi Behnagh, Navjot Kaur, Foad Kazemi, Shemsu Kedir, Mohamad Mehdi Khadembashiri, Mohammad Amin Khadembashiri, Yousef Saleh Khader, Himanshu Khajuria, Mohammad Jobair Khan, Moien AB Khan, Mahammed Ziauddin Khan Suheb, Haitham Khatatbeh, Moawiah Mohammad Khatatbeh, Sorour Khateri, Hamid Reza Khayat Kashani, Mohammad Saeid Khonji, Jagdish Khubchandani, Saeid Kian, Adnan Kisa, Aiggan Tamene Kitila, Ali-Asghar Kolahi, Hamid Reza Koohestani, Oleksii Korzh, Karel Kostev, Ashwin Laxmikant Kotnis, Ai Koyanagi, Kewal Krishan, Mohammed Kuddus, Narinder Kumar, Maria Dyah Kurniasari, Muhammad Awwal Ladan, Chandrakant Lahariya, Tri Laksono, Tea Lallukka, Iván Landires, Savita Lasrado, Basira Kankia Lawal, Thao Thi Thu Le, Trang Diep Thanh Le, Munjae Lee, Wei-Chen Lee, Yo Han Lee, Temesgen L. Lerango, David Lim, Stephen S. Lim, Giancarlo Lucchetti, Zheng Feei Ma, Azzam A. Maghazachi, Nastaran Maghbouli, Elaheh Malakan Rad, Armaan Malhotra, Ahmad Azam Malik, Mohammad Ali Mansournia, Lorenzo Giovanni Mantovani, Emmanuel Manu, Yasith Mathangasinghe, Antonio Mazzotti, Steven M. McPhail, Belayneh Mengist, Mohamed Kamal Mesregah, Tomislav Mestrovic, Ted R. Miller, Le Huu Nhat Minh, Mohammad Mirahmadi Eraghi, Erkin M. Mirrakhimov, Awoke Misganaw, Hashem Mohamadian, Ashraf Mohamadkhani, Nouh Saad Mohamed, Esmaeil Mohammadi, Soheil Mohammadi, Mesud Mohammed, Hoda Mojiri-Forushani, Ali H. Mokdad, Kaveh Momenzadeh, Sara Momtazmanesh, Lorenzo Monasta, Fateme Montazeri, Yousef Moradi, Shane Douglas Morrison, Ebrahim Mostafavi, Parsa Mousavi, Seyed Ehsan Mousavi, Admir Mulita, Efrén Murillo-Zamora, Ghulam Mustafa, Sathish Muthu, Ganesh R. Naik, Mukhammad David Naimzada, Noureddin Nakhostin Ansari, Sreenivas Narasimha Swamy, Shumaila Nargus, Paulo R.C. Nascimento, Amirreza Naseri, Zuhair S. Natto, Muhammad Naveed, Biswa Prakash Nayak, Athare Nazri-Panjaki, Mohammad Negaresh, Hadush Negash, Seyed Aria Nejadghaderi, Dang H. Nguyen, Hau Thi Hien Nguyen, Hien Quang Nguyen, Phat Tuan Nguyen, Van Thanh Nguyen, Robina Khan Niazi, Akinyemi O.D. Ofakunrin, Hassan Okati-Aliabad, Osaretin Christabel Okonji, Matthew Idowu Olatubi, Mohammad Mehdi Ommati, Michal Ordak, Mayowa O. Owolabi, Mahesh P A, Jagadish Rao Padubidri, Feng Pan, Ioannis Pantazopoulos, Seoyeon Park, Jay Patel, Shankargouda Patil, Shrikant Pawar, Paolo Pedersini, Prince Peprah, Simone Perna, Ionela-Roxana Petcu, Fanny Emily Petermann-Rocha, Hoang Tran Pham, Manon Pigeolet, Elton Junio Sady Prates, Fakher Rahim, Zahra Rahimi, Shahram Rahimi-Dehgolan, Vafa Rahimi-Movaghar, Mohammad Hifz Ur Rahman, Masoud Rahmati, Shakthi Kumaran Ramasamy, Premkumar Ramasubramani, Deepthi Rapaka, Sina Rashedi, Vahid Rashedi, Mohammad-Mahdi Rashidi, Ashkan Rasouli-Saravani, Salman Rawaf, Murali Mohan Rama Krishna Reddy, Elrashdy Moustafa Mohamed Redwan, Nazila Rezaei, Negar Rezaei, Nima Rezaei, Zahed Rezaei, Abanoub Riad, Leonardo Roever, Sharareh Roshanzamir, Priyanka Roy, Guilherme de Andrade Ruela, Aly M.A. Saad, Basema Saddik, Farideh Sadeghian, Umar Saeed, Azam Safary, Amene Saghazadeh, Dominic Sagoe, Fatemeh Saheb Sharif-Askari, Narjes Saheb Sharif-Askari, Amirhossein Sahebkar, Joseph W. Sakshaug, Afeez Abolarinwa Salami, Mohamed A. Saleh, Sana Salehi, Sara Samadzadeh, Yoseph Leonardo Samodra, Vijaya Paul Samuel, Djanilson B. Santos, Milena M. Santric-Milicevic, Muhammad Arif Nadeem Saqib, Aswini Saravanan, Susan Sawyer, Benedikt Michael Schaarschmidt, Sabyasachi Senapati, Yashendra Sethi, Allen Seylani, Amir Shafaat, Mahan Shafie, Saeed Shahabi, Ataollah Shahbandi, Shayan Shahrokhi, Masood Ali Shaikh, Muhammad Aaqib Shamim, Mohammad Ali Shamshirgaran, Sadaf Sharfaei, Amin Sharifan, Azam Sharifi, Rajendra Sharma, Saurab Sharma, Bereket Beyene Shashamo, Linhong Shi, Mika Shigematsu, Rahman Shiri, Velizar Shivarov, Emmanuel Edwar Siddig, Ehsan Sinaei, Ambrish Singh, Jasvinder A. Singh, Paramdeep Singh, Surjit Singh, Shweta Singla, Md Shahjahan Siraj, Anna Aleksandrovna Skryabina, Ranjan Solanki, Yonatan Solomon, Antonina V. Starodubova, Chandan Kumar Swain, Stella Talic, Nathan Y. Tat, Mohamad-Hani Temsah, Dufera Rikitu Terefa, Riki Tesler, Rekha Thapar, Samar Tharwat, Rasiah Thayakaran, Jansje Henny Vera Ticoalu, Marcos Roberto Tovani-Palone, Biruk Shalmeno Tusa, Sree Sudha Ty, Aniefiok John Udoakang, Seyed Mohammad Vahabi, Rohollah Valizadeh, Jef Van den Eynde, Shoban Babu Varthya, Tommi Juhani Vasankari, Narayanaswamy Venketasubramanian, Jorge Hugo Villafañe, Vasily Vlassov, Anh Truc Vo, Linh Gia Vu, Yuan-Pang Wang, Taweewat Wiangkham, Nuwan Darshana Wickramasinghe, Andrea Sylvia Winkler, Ai-Min Wu, Ali Yadollahpour, Galal Yahya, Naohiro Yonemoto, Yuyi You, Mustafa Z. Younis, Fathiah Zakham, Moein Zangiabadian, Armin Zarrintan, Chenwen Zhong, Hengxing Zhou, Zhaochen Zhu, Magdalena Zielińska, Yossef Teshome Zikarg, Osama A. Zitoun, Mohammad Zoladl, Lai-Shan Tam, and Dongze Wu.

## Supplemental information

Document S1. Figures S1‒S6

Data S1. Additional data, including Tables S1–S17 and Figures S7–S13, related to Figures 1, 2, 3, 4, and 5

Data S2. GBD 2019 MSK in Adolescents authors and affiliations

Document S2. Article plus supplemental information
