# Supplementary material for: Global pattern, trend, and cross-country inequality of early musculoskeletal disorders from 1990 to 2019, with projection from 2020 to 2050
Source: Med. 2024 Aug 9;5(8):943–962.e6. doi: 10.1016/j.medj.2024.04.009 (PMC11321819; doi:10.1016/j.medj.2024.04.009)
Supplement: Document S1. Figures S1‒S6 [file mmc1.pdf]

Med, Volume *5*

Supplemental information

Global pattern, trend, and cross-country inequality of early musculoskeletal disorders from 1990 to 2019, with projection from 2020 to 2050

GBD 2019 MSK in Adolescents Collaborators

Supplemental materials

Global pattern, trend, and cross-country inequality of early musculoskeletal disorders from 1990 to 2019, with projection from 2020 to 2050

Table of contents

sFigure 1. Number and age-specific rate of disability-adjusted life year among adolescents and young adults globally by the six musculoskeletal disorders, 1990-2019, Related to Figure 1

sFigure 2. Number and age-specific rate of prevalence, disability-adjusted life year, years lived with disability on six musculoskeletal disorders among adolescents and young adults globally by gender in 1990 and 2019, Related to Figure 2

sFigure 3. Global map of age-specific rate of prevalence for overall musculoskeletal disorders among adolescents and young adults in 2019, Related to Figure 1

sFigure 4. Global map of age-specific rate of prevalence for six musculoskeletal disorders among adolescents and young adults in 2019, Related to Figure 1

sFigure 5. Global map of age-specific rate of disability-adjusted life year for overall musculoskeletal disorders among adolescents and young adults in 2019, Related to Figure 1

sFigure 6. Global map of age-specific rate of prevalence for six musculoskeletal disorders among adolescents and young adults in 2019, Related to Figure 1

A

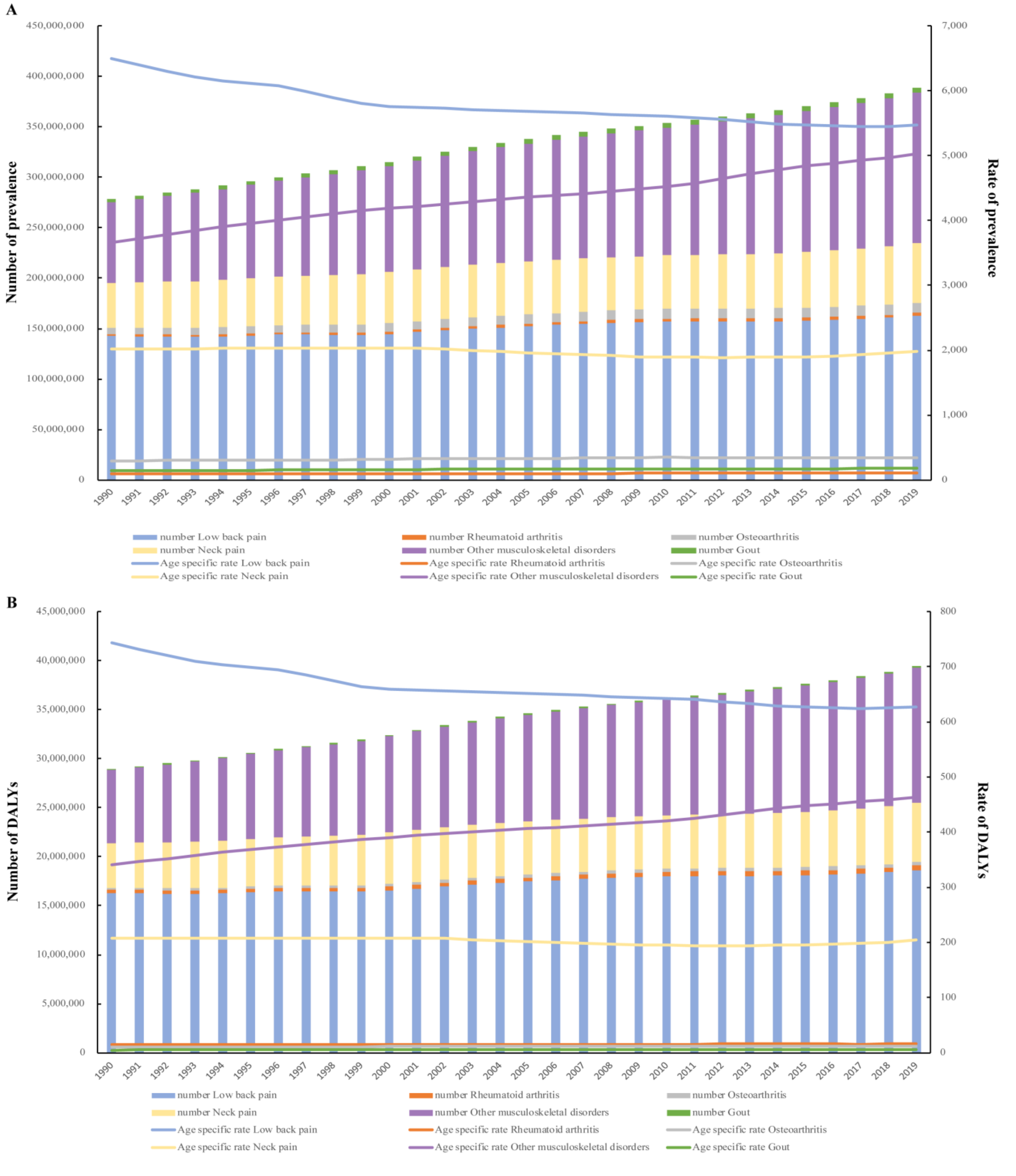

sFigure 1. Number and age-specific rate of disability-adjusted life year among adolescents and young adults globally by the six musculoskeletal disorders, 1990-2019, Related to Figure 1

A, Number of DALYs; B, age-specific rate of DALYs.

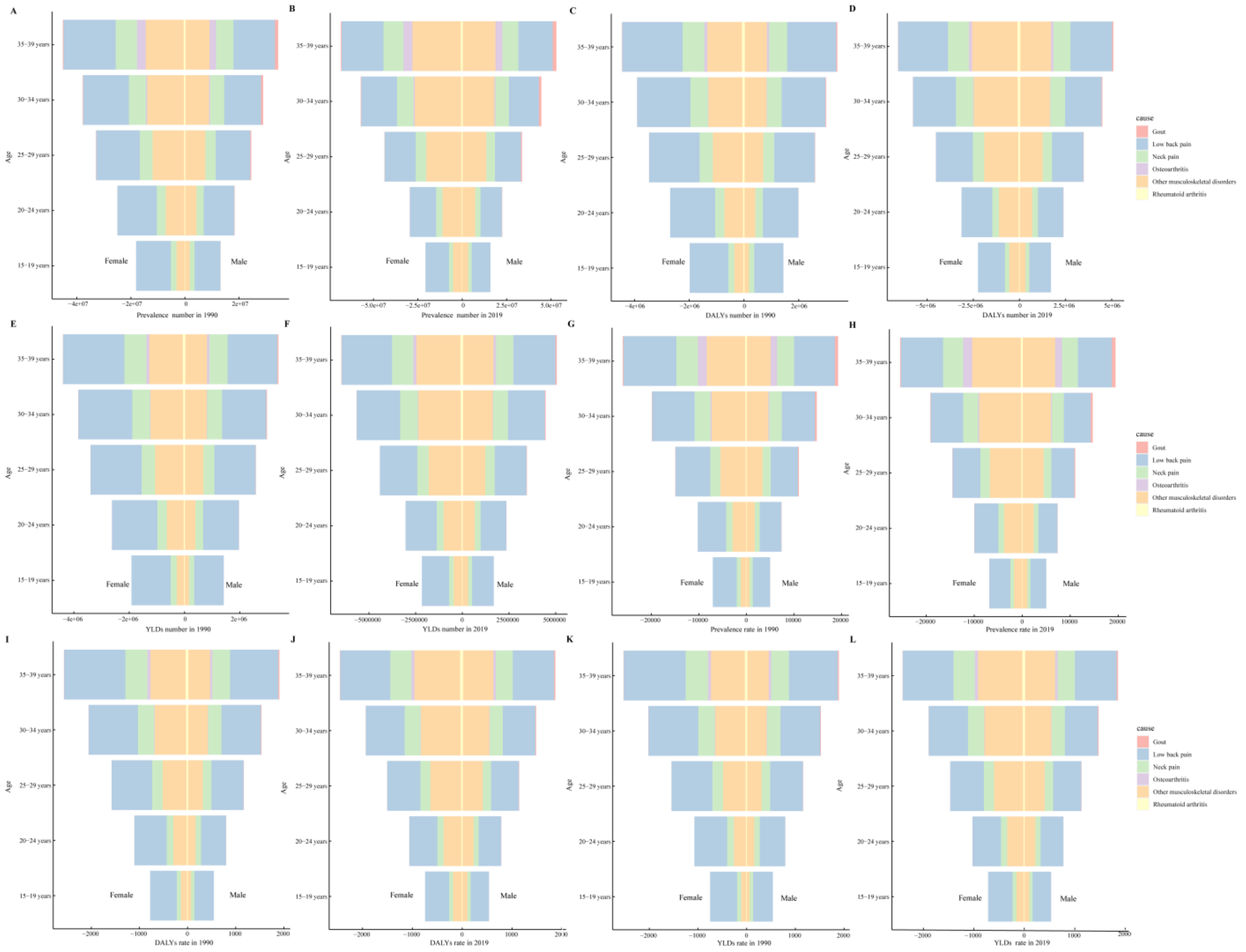

sFigure 2. Number and age-specific rate of prevalence, disability-adjusted life year, years lived with disability on six musculoskeletal disorders among adolescents and young adults globally by gender in 1990 and 2019, Related to Figure 2

A, Number of prevalence in 1990; B, Number of prevalence in 2019; C, Number of DALYs in 1990; D, Number of DALYs in 2019; E, Number of YLDs in 1990; F, Number of YLDs in 2019; G, Rate of prevalence in 1990; H, Rate of prevalence in 2019; I, Rate of DALYs in 1990; J, Rate of DALYs in 2019; K, Rate of YLDs in 1990; L, Rate of YLDs in 2019;

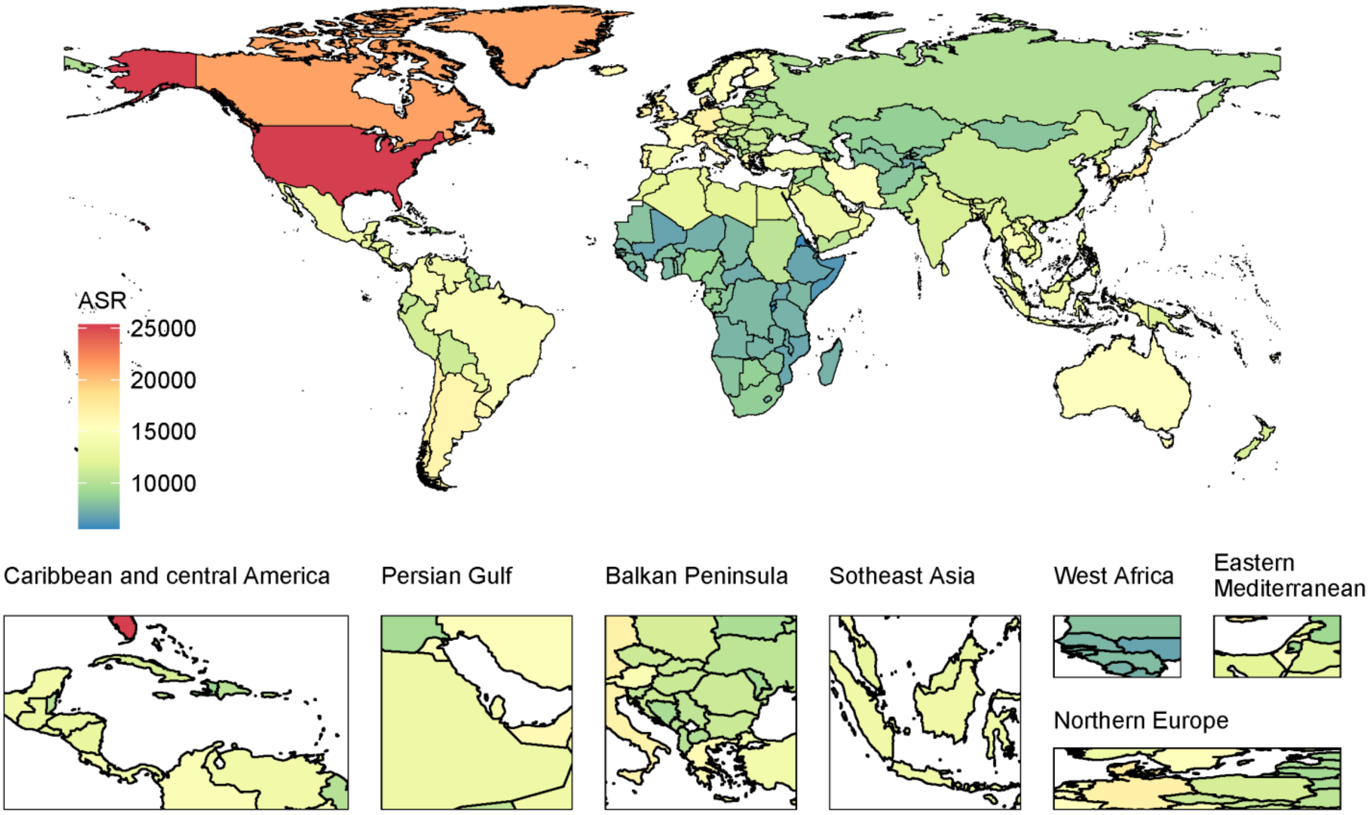

sFigure 3. Global map of age-specific rate of prevalence for overall musculoskeletal disorders among adolescents and young adults in 2019, Related to Figure 1

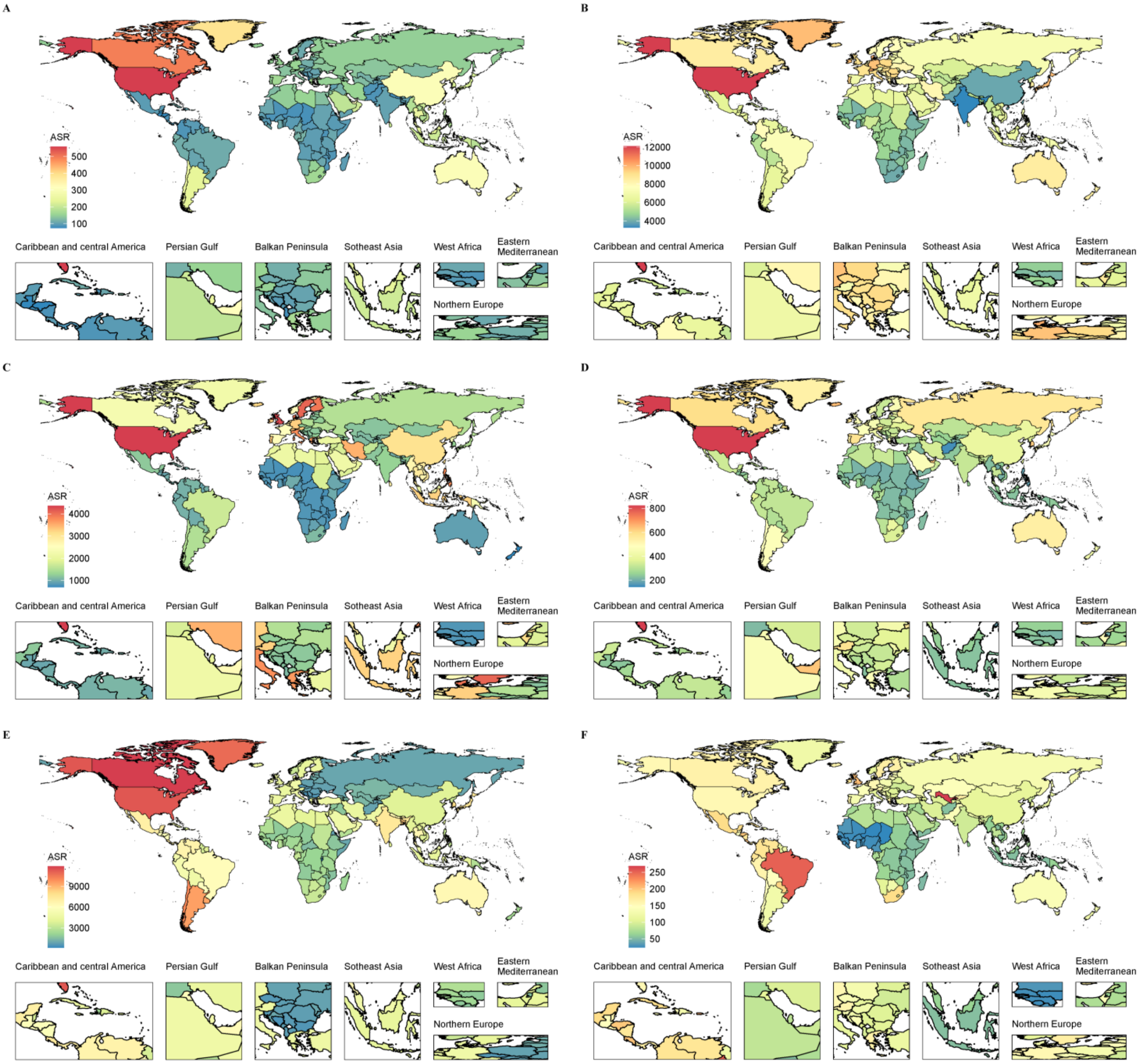

sFigure 4. Global map of age-specific rate of prevalence for six musculoskeletal disorders among adolescents and young adults in 2019, Related to Figure 1

A, Gout; B, Low back pain; C, Neck pain; D, osteoarthritis; E, other musculoskeletal disorders; F, rheumatoid arthritis

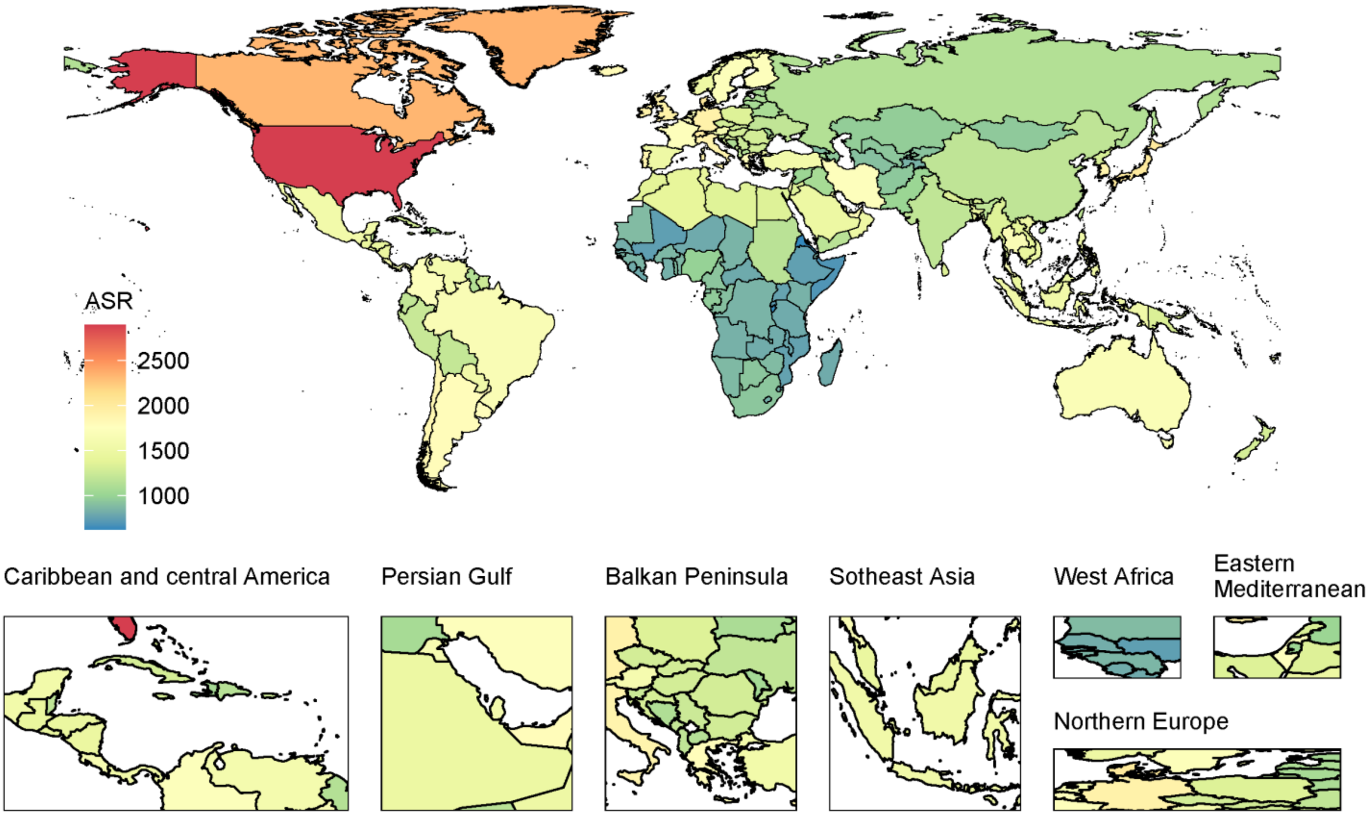

sFigure 5. Global map of age-specific rate of disability-adjusted life year for overall musculoskeletal disorders among adolescents and young adults in 2019, Related to Figure 1

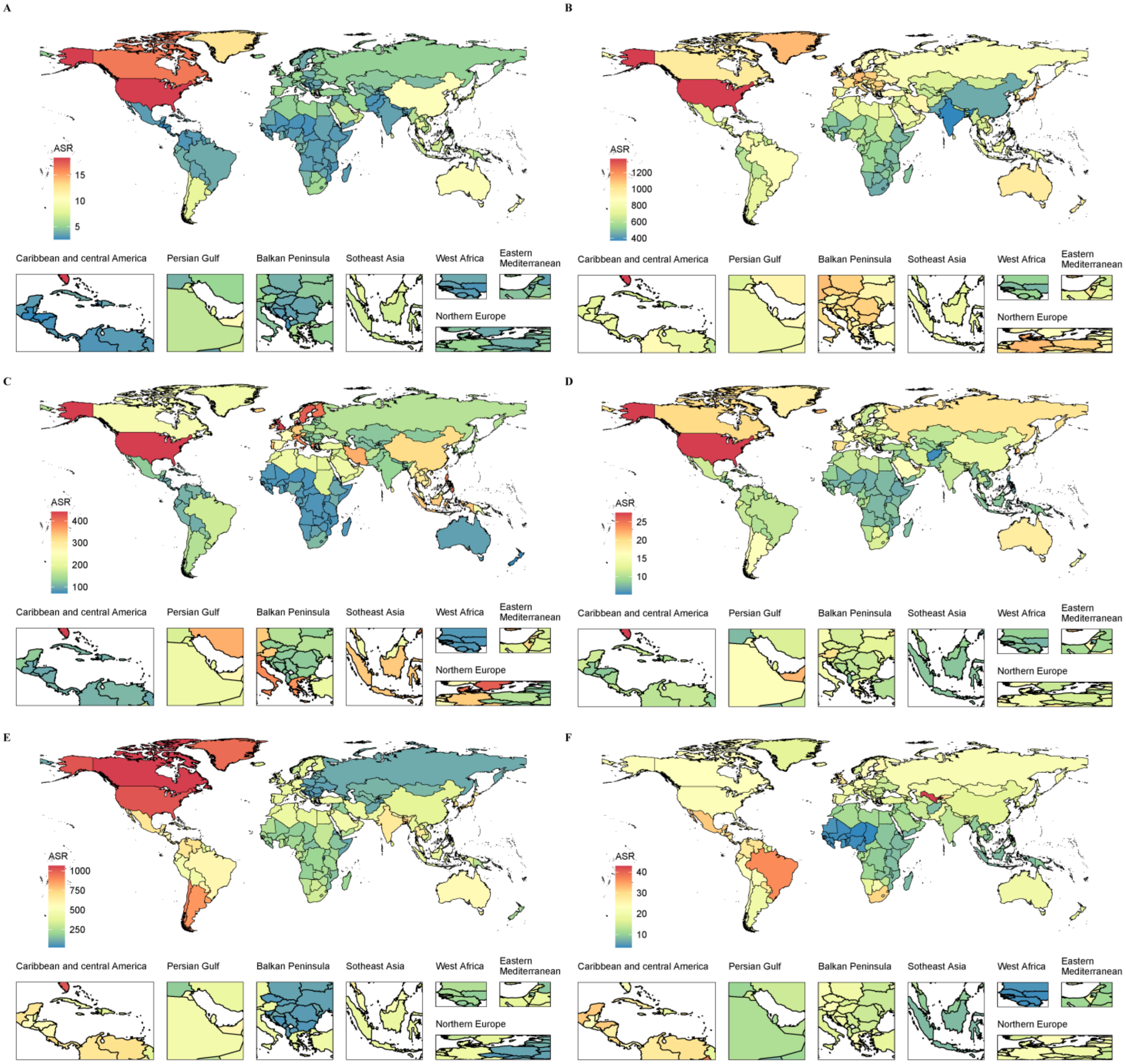

sFigure 6. Global map of age-specific rate of prevalence for six musculoskeletal disorders among adolescents and young adults in 2019, Related to Figure 1

A, Gout; B, Low back pain; C, Neck pain; D, osteoarthritis; E, other musculoskeletal disorders; F, rheumatoid arthritis
