# Supplementary material for: Global pattern, trend, and cross-country inequality of early musculoskeletal disorders from 1990 to 2019, with projection from 2020 to 2050
Source: Med. 2024 Aug 9;5(8):943–962.e6. doi: 10.1016/j.medj.2024.04.009 (PMC11321819; doi:10.1016/j.medj.2024.04.009)
Supplement: Data S2. GBD 2019 MSK in Adolescents authors and affiliations [file mmc3.pdf]

Authors

Yingzhao Jin,[1*¶] Cui Guo,[2*¶] Mohammadreza Abbasian,[3,4] Mitra Abbasifard,[5,6] Prof J Haxby Abbott,[7] Auwal Abdullahi,[8,9] Aidin Abedi,[10,11] Hassan Abidi,[12] Hassan Abolhassani,[13,14] Eman Abu-Gharbieh,[15] Prof Salahdein Aburuz,[16,17] Ahmed Abu-Zaid,[18,19] Isaac Yeboah Addo,[20,21] Oyelola A Adegboye,[22] Abiola Victor Adepoju,[23,24] Wirawan Adikusuma,[25] Qorinah Estiningtyas Sakilah Adnani,[26] Shahin Aghamiri,[27] Danish Ahmad,[28,29] Ayman Ahmed,[30,31] Prof Janardhana P Aithala,[32] Shiva Akhlaghi,[33] Sreelatha Akkala,[34] Tariq A Alalwan,[35] Prof Mohammed Albashtawy,[36] Hediyeh Alemi,[37] Prof Fadwa Alhalaiqa Naji Alhalaiqa,[38,39] Endale Alemayehu Ali,[40] Sami Almustanyir,[41,42] Rajaa M Al-Raddadi,[43] Nelson J Alvis-Zakzuk,[44,45] Prof Yaser Mohammed Al-Worafi,[46,47] Hosam Alzahrani,[48] Prof Karem H Alzoubi,[49,50] Sohrab Amiri,[51] Hubert Amu,[52] Prof Jimoh Amzat,[53,54] David B Anderson,[55] Abhishek Anil,[56,57] Benny Antony,[58] Jalal Arabloo,[59] Damelash Areda,[60,61] Al Artaman,[62] Anton A Artamonov,[63] Krishna K Aryal,[64] Prof Mohammad Asghari-Jafarabadi,[65,66] Tahira Ashraf,[67] Seyyed Shamsadin Athari,[68] Bantalem Tilaye Atinafu,[69] Maha Moh'd Wahbi Atout,[70] Sina Azadnajafabad,[71] Hamed Azhdari Tehrani,[72] Ahmed Y Azzam,[73,74] Alaa Badawi,[75,76] Nayereh Baghcheghi,[77] Ruhai Bai,[78] Vali Baigi,[79,80] Prof Maciej Banach,[81,82] Morteza Banakar,[83,84] Biswajit Banik,[85,86] Mainak Bardhan,[87] Prof Till Winfried Bärnighausen,[88,89] Hiba Jawdat Barqawi,[90] Amadou Barrow,[91,92] Azadeh Bashiri,[93] Kavita Batra,[94] Mojtaba Bayani,[95] Nebiyou Simegnew Bayileyegn,[96] Ahmet Begde,[97,98] Kebede A Beyene,[99,100] Akshaya Srikanth Bhagavathula,[101] Pankaj Bhardwaj,[102,103] Gurjit Kaur Bhatti,[104] Prof Jasvinder Singh Bhatti,[105] Rajbir Bhatti,[106] Ali Bijani,[107] Veera R Bitra,[108] Javier Brazo-Sayavera,[109] Prof Rachelle Buchbinder,[110,111] Katrin Burkart,[112,113] Yasser Bustanji,[114,115] Muhammad Hammad Butt,[116] Prof Luis Alberto Cámera,[117,118] Prof Felix Carvalho,[119] Vijay Kumar Chattu,[120,121] Akhilanand Chaurasia,[122] Guangjin Chen,[123,124] Haowei Chen,[125] Lingxiao Chen,[126] Steffan Wittrup McPhee Christensen,[127,128] Dinh-Toi Chu,[129] Isaac Sunday Chukwu,[130] Josielli Comachio,[131] Prof Natália Cruz-Martins,[132,133] Sarah Cuschieri,[134] Sriharsha Dadana,[135] Omid Dadras,[136,137] Xiaochen Dai,[112,113] Zhaoli Dai,[138,139] Saswati Das,[140] Mohsen Dashti,[141] Ivan Delgado-Enciso,[142,143] Biniyam

Demisse,[144] Edgar Denova-Gutiérrez,[145] Belay Desye,[146] Syed Masudur Rahman Dewan,[147,148] Sameer Dhingra,[149] Mengistie Diress,[150] Thanh Chi Do,[151] Thao Huynh Phuong Do,[152] Khanh Duy Khanh Doan,[153] Sulagna Dutta,[154,155] Arkadiusz Marian Dziedzic,[156] Hisham Atan Edinur,[157] Michael Ekholuenetale,[158,159] Muhammed Elhadi,[160] Sharareh Eskandarieh,[161] Francesco Esposito,[162] Adeniyi Francis Fagbamigbe,[158,163] Parisa Farokh,[164] Ali Fatehizadeh,[165] Alireza Feizkhah,[166] Ginenus Fekadu,[167,168] Prof Nuno Ferreira,[169] Getahun Fetensa,[170] Florian Fischer,[171] Prof Behzad Foroutan,[172] Masoumeh Foroutan Koudehi,[173] Prof Richard Charles Franklin,[174] Takeshi Fukumoto,[175] Aravind P Gandhi,[176] Balasankar Ganesan,[177] Shuo-Yan Gau,[178] Prof Rupesh K Gautam,[179] Abadi Kahsu Gebre,[180,181] Miglas W W Gebregergis,[182] Bardiya Ghaderi Yazdi,[183] Ali Gholami,[184,185] Tiffany K Gill,[186] Pouya Goleij,[187,188] Prof Mansueto Gomes-Neto,[189] Anmol Goyal,[190] Simon Matthew Graham,[191,192] Bin Guan,[193] Bhawna Gupta,[194] Indarchand Ratanlal Gupta,[195,196] Sapna Gupta,[197] Veer Bala Gupta,[198] Prof Vivek Kumar Gupta,[199] Farrokh Habibzadeh,[200] Wase Benti Hailu,[201] Ramtin Hajibeygi,[202] Prof Rabih Halwani,[15,203] Josep Maria Haro,[204,205] Prof Jan Hartvigsen,[206,207] Ahmed I Hasaballah,[208] Johannes Haubold,[209,210] Prof Jeffrey J Hebert,[211,212] Mohamed I Hegazy,[213] Golnaz Heidari,[214] Mohammad Heidari,[215] Kamal Hezam,[216,217] Yuta Hiraike,[218] Hassan Hosseinzadeh,[219] Prof Mehdi Hosseinzadeh,[220,221] Amir Human Hoveidaei,[222] Chi-Jen Hsu,[223] Md Nazmul Huda,[224,225] Hong-Han Huynh,[226] Prof Bing-Fang Hwang,[227,228] Segun Emmanuel Ibitoye,[229] Adalia I Ikiroma,[230] Irena M Ilic,[231] Prof Milena D Ilic,[232] Arad Iranmehr,[233] Sheikh Mohammed Shariful Islam,[234,235] Prof Nahlah Elkudssiah Ismail,[236,237] Prof Hiroyasu Iso,[238] Masao Iwagami,[239,240] Assefa N Iyasu,[241] Louis Jacob,[242,243] Prof Abdollah Jafarzadeh,[244,245] Kasra Jahankhani,[246] Nityanand Jain,[247] Ammar Abdulrahman Jairoun,[248,249] Prof Balamurugan Janakiraman,[250] Umesh Jayarajah,[251,252] Prof Shubha Jayaram,[253] Jayakumar Jeganathan,[254] Mohammad Jokar,[255,256] Prof Jost B Jonas,[257,258] Tamas Joo,[259,260] Nitin Joseph,[261] Charity Ehimwenma Joshua,[262] Gebisa Guyasa Kabito,[263] Vineet Kumar Kamal,[264,265] Himal Kandel,[266,267] Rami S Kantar,[268,269] Jafar Karami,[270,271] Ibraheem M Karaye,[272,273] Arman Karimi Behnagh,[274,275] Navjot Kaur,[276] Foad Kazemi,[277] Shemsu Kedir,[278] Mohamad Mehdi

Khadembashiri,[279,280] Mohammad Amin Khadembashiri,[281,282] Prof Yousef Saleh Khader,[283] Himanshu Khajuria,[284] Mohammad Jobair Khan,[9] Moien AB Khan,[285,286] Mahammed Ziauddin Khan suheb,[287] Haitham Khatatbeh,[288] Moawiah Mohammad Khatatbeh,[289] Sorour Khateri,[290] Hamid Reza Khayat Kashani,[291] Mohammad Saeid Khonji,[292] Prof Jagdish Khubchandani,[293] Saeid Kian,[294] Prof Adnan Kisa,[295,296] Aiggan Tamene Kitila,[297,298] Ali-Asghar Kolahi,[299] Hamid Reza Koohestani,[300] Prof Oleksii Korzh,[301] Prof Karel Kostev,[302,303] Ashwin Laxmikant Kotnis,[304] Ai Koyanagi,[305] Prof Kewal Krishan,[306] Prof Mohammed Kuddus,[307] Prof Narinder Kumar,[308] Maria Dyah Kurniasari,[309,310] Muhammad Awwal Ladan,[311] Prof Chandrakant Lahariya,[312,313] Tri Laksono,[314,315] Prof Tea Lallukka,[316] Prof Iván Landires,[317,318] Savita Lasrado,[319] Basira Kankia Lawal,[320] Thao Thi Thu Le,[321] Trang Diep Thanh Le,[321,322] Munjae Lee,[323,324] Wei-Chen Lee,[325] Prof Yo Han Lee,[326] Temesgen L Lerango,[327] David Lim,[328,329] Prof Stephen S Lim,[112,113] Prof Giancarlo Lucchetti,[330] Zheng Feei Ma,[331] Prof Azzam A Maghazachi,[90] Nastaran Maghbouli,[332] Prof Elaheh Malakan Rad,[333] Armaan K Malhotra,[334] Ahmad Azam Malik,[335,336] Mohammad Ali Mansournia,[80] Prof Lorenzo Giovanni Mantovani,[337,338] Emmanuel Manu,[52] Yasith Mathangasinghe,[339,340] Antonio Mazzotti,[341,342] Prof Steven M McPhail,[343,344] Belayneh Mengist,[345] Mohamed Kamal Mesregah,[346] Tomislav Mestrovic,[347,112] Ted R Miller,[348,349] Le Huu Nhat Minh,[350,351] Mohammad Mirahmadi Eraghi,[352,353] Prof Erkin M Mirrakhimov,[354,355] Awoke Misganaw,[113,356] Hashem Mohamadian,[357] Ashraf Mohamadkhani,[358] Nouh Saad Mohamed,[359,360] Esmaeil Mohammadi,[279,361] Soheil Mohammadi,[362] Mesud Mohammed,[363] Hoda Mojiri-forushani,[364] Ali H Mokdad,[112,113] Kaveh Momenzadeh,[3] Sara Momtazmanesh,[362,71] Lorenzo Monasta,[365] Fateme Montazeri,[71,366] Yousef Moradi,[367] Shane Douglas Morrison,[368] Ebrahim Mostafavi,[369,370] Parsa Mousavi,[371] Seyed Ehsan Mousavi,[372,373] Admir Mulita,[374] Efrén Murillo-Zamora,[375,376] Prof Ghulam Mustafa,[377,378] Sathish Muthu,[379,380] Ganesh R Naik,[381,382] Mukhammad David Naimzada,[383,384] Prof Noureddin Nakhostin Ansari,[385] Prof Sreenivas Narasimha Swamy,[386] Shumaila Nargus,[336,336] Paulo R C Nascimento,[387] Amirreza Naseri,[388] Zuhair S Natto,[389,390] Muhammad Naveed,[391] Biswa Prakash Nayak,[284] Athare Nazri-Panjaki,[392] Mohammad Negaresh,[393,394] Hadush Negash,[395] Seyed Aria

Nejadghaderi,[366,396] Dang H Nguyen,[397,398] Hau Thi Hien Nguyen,[399,400] Hien Quang Nguyen,[401] Phat Tuan Nguyen,[402] Van Thanh Nguyen,[403] Robina Khan Niazi,[404] Akinyemi O D Ofakunrin,[405,406] Hassan Okati-Aliabad,[407] Osaretin Christabel Okonji,[408] Matthew Idowu Olatubi,[409] Mohammad Mehdi Ommati,[410] Michal Ordak,[411] Prof Mayowa O Owolabi,[412,413] Prof Mahesh Padukudru P A,[414] Jagadish Rao Padubidri,[415] Feng Pan,[58] Ioannis Pantazopoulos,[416,417] Seoyeon Park,[418] Jay Patel,[419,420] Prof Shankargouda Patil,[421,422] Shrikant Pawar,[423] Paolo Pedersini,[424] Prince Peprah,[425] Prof Simone Perna,[426] Ionela-Roxana Petcu,[427] Fanny Emily Petermann-Rocha,[428,429] Hoang Tran Pham,[430] Manon Pigeolet,[431,432] Elton Junio Sady Prates,[433] Prof Fakher Rahim,[434,435] Zahra Rahimi,[436,437] Shahram Rahimi-Dehgolan,[332] Prof Vafa Rahimi-Movaghar,[79] Mohammad Hifz Ur Rahman,[438] Prof Masoud Rahmati,[439] Shakthi Kumaran Ramasamy,[440] Premkumar Ramasubramani,[441] Deepthi Rapaka,[442] Sina Rashedi,[443,396] Vahid Rashedi,[444] Mohammad-Mahdi Rashidi,[71,299] Ashkan Rasouli-Saravani,[445] Prof Salman Rawaf,[446,447] Murali Mohan Rama Krishna Reddy,[448] Prof Elrashdy Moustafa Mohamed Redwan,[449,450] Nazila Rezaei,[71] Negar Rezaei,[71,451] Prof Nima Rezaei,[13,452] Zahed Rezaei,[453] Abanoub Riad,[454,455] Leonardo Roever,[456] Sharareh Roshanzamir,[457,458] Priyanka Roy,[459] Prof Guilherme de Andrade Ruela,[460,461] Prof Aly M A Saad,[462] Basema Saddik,[463] Farideh Sadeghian,[464,79] Prof Umar Saeed,[465,466] Azam Safary,[467] Amene Saghazadeh,[13] Dominic Sagoe,[468] Fatemeh Saheb Sharif-Askari,[469] Narjes Saheb Sharif-Askari,[15] Amirhossein Sahebkar,[470,471] Joseph W Sakshaug,[472,473] Afeez Abolarinwa Salami,[474,475] Mohamed A Saleh,[203,476] Sana Salehi,[477] Sara Samadzadeh,[478,479] Yoseph Leonardo Samodra,[480,480] Prof Vijaya Paul Samuel,[481] Prof Djanilson B Santos,[482] Prof Milena M Santric-Milicevic,[231,483] Muhammad Arif Nadeem Saqib,[484,485] Aswini Saravanan,[486,487] Prof Susan M Sawyer,[488,489] Prof Benedikt Michael Schaarschmidt,[209] Sabyasachi Senapati,[490] Yashendra Sethi,[491] Allen Seylani,[492] Amir Shafaat,[493] Mahan Shafie,[494] Saeed Shahabi,[84] Ataollah Shahbandi,[495] Shayan Shahrokhi,[496,497] Masood Ali Shaikh,[498] Muhammad Aaqib Shamim,[56] Mohammad Ali Shamshirgaran,[499] Sadaf Sharfaei,[500,501] Amin Sharifan,[502,503] Azam Sharifi,[504] Prof Rajendra Sharma,[505] Saurab Sharma,[506,507] Bereket Beyene Shashamo,[144] Linhong Shi,[1] Mika Shigematsu,[508] Rahman Shiri,[509] Velizar

Shivarov,[510,511] Emmanuel Edwar Siddig,[512,513] Ehsan Sinaei,[514] Ambrish Singh,[58] Prof Jasvinder A Singh,[515,516] Paramdeep Singh,[517] Surjit Singh,[56] Shweta Singla,[518] Md Shahjahan Siraj,[519] Anna Aleksandrovna Skryabina,[520] Ranjan Solanki,[521,522] Yonatan Solomon,[523] Antonina V Starodubova,[524,525] Chandan Kumar Swain,[526] Stella Talic,[527] Nathan Y Tat,[528,529] Mohamad-Hani Temsah,[530] Dufera Rikitu Terefa,[531,201] Riki Tesler,[532] Rekha Thapar,[261] Samar Tharwat,[533] Rasiah Thayakaran,[534,535] Jansje Henny Vera Ticoalu,[536] Marcos Roberto Tovani-Palone,[537,538] Biruk Shalmeno Tusa,[539] Sree Sudha Ty,[540] Aniefiok John Udoakang,[541] Seyed Mohammad Vahabi,[361] Rohollah Valizadeh,[542] Jef Van den Eynde,[543] Shoban Babu Varthya,[56] Prof Tommi Juhani Vasankari,[544,545] Prof Narayanaswamy Venketasubramanian,[546,547] Jorge Hugo Villafañe,[424] Prof Vasily Vlassov,[548] Anh Truc Vo,[112] Linh Gia Vu,[549,399] Yuan-Pang Wang,[550] Taweewat Wiangkham,[551] Nuwan Darshana Wickramasinghe,[552] Prof Andrea Sylvia Winkler,[553,554] Prof Ai-Min Wu,[555] Ali Yadollahpour,[556] Galal Yahya,[557,558] Naohiro Yonemoto,[559,560] Yuyi You,[561,266] Prof Mustafa Z Younis,[562,563] Fathiah Zakham,[564,565] Moein Zangiabadian,[366] Armin Zarrintan,[141] Chenwen Zhong,[566] Hengxing Zhou,[567] Zhaochen Zhu,[568] Magdalena Zielińska,[569] Yossef Teshome Zikarg,[570] Osama A Zitoun,[571,572] Mohammad Zoladl,[573] Prof Lai-shan Tam,[1#¶] Dongze Wu[574#¶].

[*] Joint first authors

[#] Joint senior authors

[¶] Writing authors

Author affiliations

[1]Department of Medicine & Therapeutics, The Chinese University of Hong Kong, Hong Kong, China; [2]Department of Urban Planning and Design, University of Hong Kong, Hong Kong, China; [3]Department of Orthopedic Surgery, Harvard University, Boston, MA, USA; [4]Department of Orthopaedic Surgery, Shahid Beheshti University of Medical Sciences, Tehran, Tehran, Iran; [5]Department of Internal Medicine, Rafsanjan University of Medical Sciences, Rafsanjan, Iran; [6]Clinical Research

Development Unit, Rafsanjan University of Medical Sciences, Rafsanjan, Iran; [7]Centre for Musculoskeletal Outcomes Research, Department of Surgical Sciences, Otago Medical School, University of Otago, Dunedin, New Zealand; [8]Department of Physiotherapy, Bayero University Kano, Kano, Nigeria; [9]Department of Rehabilitation Sciences, Hong Kong Polytechnic University, Hong Kong, China; [10]Department of Neurosurgery, University of Southern California, Los Angeles, CA, USA; [11]Keck School of Medicine, University of Southern California, Los Angeles, CA, USA; [12]Laboratory Technology Sciences Department, Yasuj University of Medical Sciences, Yasuj, Iran; [13]Research Center for Immunodeficiencies, Tehran University of Medical Sciences, Tehran, Iran; [14]Department of Medical Biochemistry and Biophysics, Karolinska Institute, Stockholm, Sweden; [15]Clinical Sciences Department, University of Sharjah, Sharjah, United Arab Emirates; [16]Department of Therapeutics, United Arab Emirates University, Al Ain, United Arab Emirates; [17]College of Pharmacy, University of Jordan, Amman, Jordan; [18]Department of Surgery, Alfaisal University, Riyadh, Saudi Arabia; [19]College of Graduate Health Sciences, University of Tennessee, Memphis, TN, USA; [20]Centre for Social Research in Health, University of New South Wales, Sydney, NSW, Australia; [21]Quality and Systems Performance Unit, Cancer Institute NSW, Sydney, NSW, Australia; [22]Menzies School of Health Research, Charles Darwin University, Darwin, NT, Australia; [23]HIV and Infectious Diseases Department, Jhpiego, Abuja, Nigeria; [24]Adolescent Research and Care, Adolescent Friendly Research Initiative and Care, Ado Ekiti, Nigeria; [25]Department of Pharmacy, University of Muhammadiyah Mataram, Mataram, Indonesia; [26]Faculty of Medicine, Universitas Padjadjaran (Padjadjaran University), Bandung, Indonesia; [27]Department of Biotechnology, Shahid Beheshti University of Medical Sciences, Tehran, Iran; [28]School of Medicine and Psychology, Australian National University, Canberra, ACT, Australia; [29]Public Health Foundation of India, Gandhinagar, India; [30]Institute of Endemic Diseases, University of Khartoum, Khartoum, Sudan; [31]Swiss Tropical and Public Health Institute, University of Basel, Basel, Switzerland; [32]Department of Orthopedics, Yenepoya Medical College, Mangalore, India; [33]Department of Biomolecular Science, University of Mississippi, Oxford, USA; [34]Department of

Management, Policy, and Community Health, University of Texas, Houston, TX, USA; [35]Department of Biology, University of Bahrain, Sakhir, Bahrain; [36]Community and Mental Health Department, Al al-Bayt University, Mafraq, Jordan; [37]Hematology, Oncology and Stem Cell Transplantation Research Center, Tehran University of Medical Sciences, Tehran, Iran; [38]College of Nursing, Qatar University, Doha, Qatar; [39]Psychological Sciences Association, Amman, Jordan; [40]Department of Public Health and Primary Care, Katholieke Universiteit Leuven, Leuven, Belgium; [41]College of Medicine, Alfaisal University, Riyadh, Saudi Arabia; [42]Ministry of Health, Riyadh, Saudi Arabia; [43]Department of Community Medicine, King Abdulaziz University, Jeddah, Saudi Arabia; [44]Department of Economic Sciences, Universidad de la Costa (University of the Coast), Barranquilla, Colombia; [45]National Health Observatory, National Institute of Health, Bogota, Colombia; [46]Department of Medical Sciences, Azal University for Human Development, Sana'a, Yemen; [47]Department of Clinical Sciences, University of Science and Technology of Fujairah, Fujairah, United Arab Emirates; [48]Department of Physiotherapy, Taif University, Taif, Saudi Arabia; [49]Department of Pharmacy Practice and Pharmacotherapeutics, University of Sharjah, Sharjah, United Arab Emirates; [50]Department of Clinical Pharmacy, Jordan University of Science and Technology, Irbid, Jordan; [51]Medicine, Quran and Hadith Research Center, Baqiyatallah University of Medical Sciences, Tehran, Iran; [52]Department of Population and Behavioural Sciences, University of Health and Allied Sciences, Ho, Ghana; [53]Department of Sociology, Usmanu Danfodiyo University, Sokoto, Sokoto, Nigeria; [54]Department of Sociology, University of Johannesburg, Johannesburg, South Africa; [55]Faculty of Medicine, University of Sydney, Sydney, NSW, Australia; [56]Department of Pharmacology, All India Institute of Medical Sciences, Jodhpur, India; [57]All India Institute of Medical Sciences, Bhubaneswar, India; [58]Menzies Institute for Medical Research, University of Tasmania, Hobart, TAS, Australia; [59]Health Management and Economics Research Center, Iran University of Medical Sciences, Tehran, Iran; [60]Ottawa University, Surprise, AZ, USA; [61]School of Life Sciences, Arizona State University, Tempe, AZ, USA; [62]Department of Health Sciences, Zayed University, Dubai, United Arab Emirates; [63]Department of Biophysics, Russian

Academy of Sciences, Moscow, Russia; [64]Monitoring Evaluation and Operational Research Project, Abt Associates Nepal, Lalitpur, Nepal; [65]Cabrini Research, Cabrini Health, Malvern, VIC, Australia; [66]School of Public Health and Preventative Medicine, Monash University, Melbourne, VIC, Australia; [67]University Institute of Radiological Sciences and Medical Imaging Technology, The University of Lahore, Lahore, Pakistan; [68]Department of Immunology, Zanjan University of Medical Sciences, Zanjan, Iran; [69]School of Nursing and Midwifery, Debre Berhan University, Debre Berhan, Ethiopia; [70]Faculty of Nursing, Philadelphia University, Amman, Jordan; [71]Non-communicable Diseases Research Center, Tehran University of Medical Sciences, Tehran, Iran; [72]Internal Medicine, Shahid Beheshti University of Medical Sciences, Tehran, Iran; [73]Department of Neurovascular Research, Nested Knowledge, Inc., Saint Paul, MN, USA; [74]Faculty of Medicine, October 6 University, 6th of October City, Egypt; [75]Public Health Risk Sciences Division, Public Health Agency of Canada, Toronto, ON, Canada; [76]Department of Nutritional Sciences, University of Toronto, Toronto, ON, Canada; [77]Department of Nursing, Saveh University of Medical Sciences, Saveh, Iran; [78]School of Public Affairs, Nanjing University of Science and Technology, Nanjing, China; [79]Sina Trauma and Surgery Research Center, Tehran University of Medical Sciences, Tehran, Iran; [80]Department of Epidemiology and Biostatistics, Tehran University of Medical Sciences, Tehran, Iran; [81]Department of Hypertension, Medical University of Lodz, Lodz, Poland; [82]Polish Mothers' Memorial Hospital Research Institute, Lodz, Poland; [83]Dental Research Center, Tehran University of Medical Sciences, Tehran, Iran; [84]Health Policy Research Center, Shiraz University of Medical Sciences, Shiraz, Iran; [85]Institute of Health and Wellbeing (IHW), Federation University Australia, Melbourne, VIC, Australia; [86]Manna Institute, University of New England, Armidale, NSW, Australia; [87]Miami Cancer Institute, Baptist Health South Florida, Miami, FL, USA; [88]Heidelberg Institute of Global Health (HIGH), Heidelberg University, Heidelberg, Germany; [89]T.H. Chan School of Public Health, Harvard University, Boston, MA, USA; [90]Department of Clinical Sciences, University of Sharjah, Sharjah, United Arab Emirates; [91]Department of Public & Environmental Health, University of The Gambia, Brikama, The Gambia; [92]Epidemiology and Disease

Control Unit, Ministry of Health, Kotu, The Gambia; [93]Health Information Management, Shiraz University of Medical Sciences, Shiraz, Iran; [94]Department of Medical Education, University of Nevada, Las Vegas, Las Vegas, NV, USA; [95]Department of Periodontics, School of Dentistry, Arak University of Medical Sciences, Arak, Iran; [96]Department of Surgery, Jimma University, Jimma, Ethiopia; [97]School of Sport, Exercise and Health Sciences, Loughborough University, Loughborough, UK; [98]School of Public Health, Imperial College London, London, UK; [99]School of Pharmacy, University of Auckland, Auckland, New Zealand; [100]Department of Pharmaceutical and Administrative Sciences, University of Health Sciences and Pharmacy in St. Louis, St louis, MO, USA; [101]Department of Health, Human Performance and Recreation, University of Arkansas, Fayetteville, AR, USA; [102]Department of Community Medicine and Family Medicine, All India Institute of Medical Sciences, Jodhpur, India; [103]School of Public Health, All India Institute of Medical Sciences, Jodhpur, India; [104]Medical Lab Technology, Chandigarh University, Mohali, India; [105]Human Genetics and Molecular Medicine, Central University of Punjab, Bathinda, India; [106]Department of Pharmaceutical Sciences, Guru Nanak Dev University, Amritsar, India; [107]Social Determinants of Health Research Center, Babol University of Medical Sciences, Babol, Iran; [108]Faculty of Health Sciences, University of Botswana, Gaborone, Botswana; [109]Centro Regional Universitario Noreste, University of the Republic of Uruguay, Rivera, Uruguay; [110]Department of Epidemiology and Preventive Medicine, Monash University, Melbourne, VIC, Australia; [111]Monash Department of Clinical Epidemiology at Cabrini Hospital, Cabrini Institute, Melbourne, VIC, Australia; [112]Institute for Health Metrics and Evaluation, University of Washington, Seattle, WA, USA; [113]Department of Health Metrics Sciences, School of Medicine, University of Washington, Seattle, WA, USA; [114]Department of Biopharmaceutics and Clinical Pharmacy, The University of Jordan, Amman, Jordan; [115]Department of Basic Biomedical Sciences, University of Sharjah, Sharjah, United Arab Emirates; [116]Faculty of Pharmacy, University of Central Punjab, Lahore, Pakistan; [117]Internal Medicine Department, Hospital Italiano de Buenos Aires (Italian Hospital of Buenos Aires), Buenos Aires, Argentina; [118]Board of Directors,

Argentine Society of Medicine, Buenos Aires, Argentina; [119]Research Unit on Applied Molecular Biosciences (UCIBIO), University of Porto, Porto, Portugal; [120]Temerty Faculty of Medicine, University of Toronto, Toronto, ON, Canada; [121]Saveetha Dental College, Saveetha University, Chennai, India; [122]Oral Medicine and Radiology, King George's Medical University, Lucknow, India; [123]Department of Stomatology, Huazhong University of Science and Technology, Wuhan, China; [124]Hubei Province Key Laboratory of Oral and Maxillofacial Development and Regeneration, Wuhan, China; [125]Clinical Research Center, Southern Medical University, Guangzhou, China; [126]Institute of Bone and Joint Research, University of Sydney, Sydney, NSW, Australia; [127]Department of Health Science and Technology, Aalborg University, Aalborg, Denmark; [128]Department of Physiotherapy, University College of Northern Denmark, Aalborg, Denmark; [129]Center for Biomedicine and Community Health, VNU-International School, Hanoi, Viet Nam; [130]Department of Paediatric Surgery, Federal Medical Centre, Umuahia, Nigeria; [131]Faculty of Medicine and Health, University of Sydney, Sydney, NSW, Australia; [132]Therapeutic and Diagnostic Technologies, Cooperativa de Ensino Superior Politécnico e Universitário (Polytechnic and University Higher Education Cooperative), Gandra, Portugal; [133]Institute for Research and Innovation in Health, University of Porto, Porto, Portugal; [134]Anatomy Department, University of Malta, Msida, Malta; [135]Department of Internal Medicine, Cheyenne regional medical center, Cheyenne, WY, USA; [136]Department of Addiction Medicine, Haukland University Hospital, Bergen, Norway; [137]Department of Global Public Health and Primary Care, University of Bergen, Bergen, Norway; [138]School of Population Health, University of New South Wales, Sydney, NSW, Australia; [139]School of Pharmacy and Charles Perkins Centre, University of Sydney, Sydney, NSW, Australia; [140]Department of Biochemistry, Ministry of Health and Welfare, New Delhi, India; [141]Department of Radiology, Tabriz University of Medical Sciences, Tabriz, Iran; [142]School of Medicine, University of Colima, Colima, Mexico; [143]Department of Research, Colima State Health Services, Colima, Mexico; [144]Department of Nursing, Arba Minch University, Arba Minch, Ethiopia; [145]Center for Nutrition and Health Research, National Institute of Public Health, Cuernavaca, Mexico; [146]Department of

Public Health, Adigrat University, Adigrat, Ethiopia; [147]Department of Pharmacy, University of Asia Pacific, Dhaka, Bangladesh; [148]Pharmacology Department, Center for Life Sciences Research Bangladesh, Dhaka, Bangladesh; [149]Department of Pharmacy Practice, National Institute of Pharmaceutical Education and Research, Hajipur, India; [150]Department of Human Physiology, University of Gondar, Gondar, Ethiopia; [151]Department of Medicine, Pham Ngoc Thach University of Medicine, Ho Chi Minh City, Viet Nam; [152]Department of Medicine, Can Tho University of Medicine and Pharmacy, Can Tho, Viet Nam; [153]Department of Pathology, Temple University Hospital, Philadelphia, PA, USA; [154]Department of Oral Biology and Biomedical Sciences, MAHSA University, Jenjarom, Malaysia; [155]Department of Medical Sciences, Bharath Institute of Higher Education and Research (BIHER), Chennai, India; [156]Department of Conservative Dentistry with Endodontics, Medical University of Silesia, Katowice, Poland; [157]School of Health Sciences, Universiti Sains Malaysia (University of Science Malaysia), Kubang Kerian, Malaysia; [158]Department of Epidemiology and Medical Statistics, University of Ibadan, Ibadan, Nigeria; [159]Faculty of Public Health, University of Ibadan, Ibadan, Nigeria; [160]Faculty of Medicine, University of Tripoli, Tripoli, Libya; [161]Multiple Sclerosis Research Center, Tehran University of Medical Sciences, Tehran, Iran; [162]Department of Biomedical and Nueromotor Sciences, University of Bologna, Bologna, Italy; [163]Research Centre for Healthcare and Community, Coventry University, Coventry, UK; [164]Department of Microbiology, Shahid Beheshti University of Medical Sciences, Tehran, Iran; [165]Department of Environmental Health Engineering, Isfahan University of Medical Sciences, Isfahan, Iran; [166]Department of Social medicine and epidemiology, Guilan University of Medical Sciences, Rasht, Iran; [167]School of Pharmacy, The Chinese University of Hong Kong, Hong Kong, China; [168]Department of Pharmacy, Wollega University, Nekemte, Ethiopia; [169]Department of Social Sciences, University of Nicosia, Nicosia, Cyprus; [170]Department of Nursing, Wollega University, Nekemte, Ethiopia; [171]Institute of Public Health, Charité Universitätsmedizin Berlin (Charité Medical University Berlin), Berlin, Germany; [172]Department of Pharmacology, Iranshahr University of Medical Sciences, Iranshahr, Iran; [173]Biomaterials and

Medicinal Chemistry Research Centre, AJA University of Medical Sciences, Tehran, Iran; [174]School of Public Health, Medical, and Veterinary Sciences, James Cook University, Douglas, QLD, Australia; [175]Department of Dermatology, Kobe University, Kobe, Japan; [176]Community Medicine, ESIC Medical College & Hospital, Hyderabad, India; [177]Institute of Health and Wellbeing, Federation University, Churchill, VIC, Australia; [178]School of Medicine, Chung Shan Medical University, Taichung, Taiwan; [179]Department of Pharmacology, Indore Institute of Pharmacy, Indore, India; [180]Nutrition and Health Innovation Research Institute, East African Community Health Research Commission, Joondalup, WA, Australia; [181]School of Pharmacy, Mekelle University, Mekelle, Ethiopia; [182]Department of Midwifery, Adigrat University, Adigrat, Ethiopia; [183]Neurology Department, Tehran University of Medical Sciences, Tehran, Iran; [184]Department of Epidemiology and Biostatistics, Neyshabur University of Medical Sciences, Neyshabur, Iran; [185]Non-communicable Diseases Research Center, Neyshabur University of Medical Sciences, Neyshabur, Iran; [186]Adelaide Medical School, University of Adelaide, Adelaide, SA, Australia; [187]Department of Genetics, Sana Institute of Higher Education, Sari, Iran; [188]Universal Scientific Education and Research Network (USERN), Kermanshah University of Medical Sciences, Kermanshah, Iran; [189]Department of Physiotherapy, Federal University of Bahia, Salvador, Brazil; [190]Blood and Marrow Transplantation and Cellular Therapy Program, Stanford University, Palo Alto, CA, USA; [191]Nuffield Department of Orthopaedics, Oxford University, Oxford, UK; [192]Liverpool Orthopaedic and Trauma Service, University of Liverpool, Liverpool, UK; [193]Cheeloo College of Medicine, Shandong University, Jinan, China; [194]Department of Public Health, Torrens University Australia, Melbourne, VIC, Australia; [195]Department of Biotechnology, Government Institute of Science, Aurangabad, India; [196]Department of Biotechnology, Sant Gadge Baba Amravati University, Amravati, India; [197]Toxicology Department, Shriram Institute for Industrial Research, Delhi, India; [198]School of Medicine, Deakin University, Geelong, VIC, Australia; [199]Faculty of Medicine Health and Human Sciences, Macquarie University, Sydney, NSW, Australia; [200]Global Virus Network, Middle East Region, Shiraz, Iran; [201]Department of Public Health, Wollega University,

Nekemte, Ethiopia; [202]Department of Radiology, Tehran University of Medical Sciences, Tehran, Iran; [203]College of Medicine, University of Sharjah, Sharjah, United Arab Emirates; [204]Research Unit, University of Barcelona, Barcelona, Spain; [205]Biomedical Research Networking Center for Mental Health Network (CiberSAM), Barcelona, Spain; [206]Department of Sports Science and Clinical Biomechanics, University of Southern Denmark, Odense, Denmark; [207]Research Department, Nordic Institute of Chiropractic and Clinical Biomechanics, Odense, Denmark; [208]Department of Zoology and Entomology, Al Azhar University, Cairo, Egypt; [209]Department of Diagnostic and Interventional Radiology and Neuroradiology, University Hospital Essen, Essen, Germany; [210]Institute of Artificial Intelligence in Medicine, University Hospital Essen, Essen, Germany; [211]Faculty of Kinesiology, University of New Brunswick, Fredericton, NB, Canada; [212]School of Psychology and Exercise Science, Murdoch University, Murdoch, WA, Australia; [213]Department of Neurology, Cairo University, Cairo, Egypt; [214]Independent Consultant, Santa Clara, CA, USA; [215]Community-Oriented Nursing Midwifery Research Center, Shahrekord University of Medical Sciences, Shahrekord, Iran; [216]Department of Microbiology, Taiz University, Taiz, Yemen; [217]School of Medicine, Nankai University, Tianjin, China; [218]Division for Health Service Promotion, University of Tokyo, Tokyo, Japan; [219]School of Health & Society, University of Wollongong, Wollongong, NSW, Australia; [220]Institute of Research and Development, Duy Tan University, Da Nang, Viet Nam; [221]Department of Computer Science, University of Human Development, Sulaymaniyah, Iraq; [222]Sinai Hospital, Baltimore, MD, USA; [223]Department of Orthopedics, The Fifth Affiliated Hospital of Sun Yat-sen University, Guangdong, China; [224]School of Clinical Medicine, University of New South Wales, Sydney, NSW, Australia; [225]Research Division, ARCED Foundation, Dhaka, Bangladesh; [226]School of Biotechnology, Tan Tao University, Long An, Viet Nam; [227]Department of Occupational Safety and Health, China Medical University, Taichung, Taiwan; [228]Department of Occupational Therapy, Asia University, Taiwan, Taichung, Taiwan; [229]Department of Health Promotion and Education, University of Ibadan, Ibadan, Nigeria; [230]Clinical Effectiveness, NHS National Services Scotland, Edinburgh, UK;

[231]Faculty of Medicine, University of Belgrade, Belgrade, Serbia; [232]Department of Epidemiology, University of Kragujevac, Kragujevac, Serbia; [233]Department of Neurosurgery, Tehran University of Medical Sciences, Tehran, Iran; [234]Institute for Physical Activity and Nutrition, Deakin University, Burwood, VIC, Australia; [235]Sydney Medical School, University of Sydney, Sydney, NSW, Australia; [236]Department of Clinical Pharmacy & Pharmacy Practice, Asian Institute of Medicine, Science and Technology, Kedah, Malaysia; [237]Malaysian Academy of Pharmacy, Puchong, Malaysia; [238]Public Health Department of Social Medicine, Osaka University, Suita, Japan; [239]Department of Health Services Research, University of Tsukuba, Tsukuba, Japan; [240]Department of Non-communicable Disease Epidemiology, London School of Hygiene & Tropical Medicine, London, UK; [241]Department of Nursing, Aksum University, Aksum, Ethiopia; [242]Research and Development Unit, Biomedical Research Networking Center for Mental Health Network (CiberSAM), Sant Boi de Llobregat, Spain; [243]Faculty of Medicine, University of Versailles Saint-Quentin-en-Yvelines, Montigny-le-Bretonneux, France; [244]Department of Immunology, Kerman University of Medical Sciences, Kerman, Iran; [245]Department of Immunology, Rafsanjan University of Medical Sciences, Rafsanjan, Iran; [246]Department of Immunology, Shahid Beheshti University of Medical Sciences, Tehran, Iran; [247]Statistics Unit, Riga Stradins University, Riga, Latvia; [248]Department of Clinical Pharmacy, University of Science Malaysia, Penang, Malaysia; [249]Department of Health and Safety, United Arab Emirates University, Dubai, United Arab Emirates; [250]SRM College of Physiotherapy, SRM Institute of Science and Technology (SRMIST), Chennai, India; [251]Postgraduate Institute of Medicine, University of Colombo, Colombo, Sri Lanka; [252]Department of Surgery, National Hospital, Colombo, Sri Lanka; [253]Department of Biochemistry, Government Medical College, Mysuru, India; [254]Department of General Medicine, Manipal Academy of Higher Education, Mangalore, India; [255]Zoonoses Research Center, Islamic Azad University, Karaj, Iran; [256]Department of Clinical Sciences, Jahrom University of Medical Sciences, Jahrom, Iran; [257]Institute of Molecular and Clinical Ophthalmology Basel, Basel, Switzerland; [258]Department of Ophthalmology, Heidelberg University,

Mannheim, Germany; [259]Health Services Management Training Centre, Semmelweis University, Budapest, Hungary; [260]Hungarian Health Management Association, Budapest, Hungary; [261]Department of Community Medicine, Manipal Academy of Higher Education, Mangalore, India; [262]Department of Economics, National Open University, Benin City, Nigeria; [263]Institute of Public Health, University of Gondar, Gondar, Ethiopia; [264]Division of Epidemiology and Biostatistics, National Institute of Epidemiology, Chennai, India; [265]Department of Biostatistics, Indian Council of Medical Research, New Delhi, India; [266]Save Sight Institute, University of Sydney, Sydney, NSW, Australia; [267]Sydney Eye Hospital, South Eastern Sydney Local Health District, Sydney, NSW, Australia; [268]The Hansjörg Wyss Department of Plastic and Reconstructive Surgery, Nab'a Al-Hayat Foundation for Medical Sciences and Health Care, New York, NY, USA; [269]Cleft Lip and Palate Surgery, Global Smile Foundation, Norwood, MA, USA; [270]Laboratory Science Department, Khomein University of Medical Sciences, Khomein, Iran; [271]Department of Immunology, Tehran University of Medical Sciences, Tehran, Iran; [272]School of Health Professions and Human Services, Hofstra University, Hempstead, NY, USA; [273]Department of Anesthesiology, Montefiore Medical Center, Bronx, NY, USA; [274]Endocrine Research Center, Iran University of Medical Sciences, Tehran, Iran; [275]Department of Echocardiography, Iran University of Medical Sciences, Tehran, Iran; [276]Department of ENT, Dr. B. R. Ambedkar State Institute of Medical Sciences (AIMS), Mohali, India; [277]Department of Neurosurgery, Johns Hopkins University, Baltimore, MD, USA; [278]Department of Public Health, Werabe University, Werabe, Ethiopia; [279]Tehran University of Medical Sciences, Tehran, Iran; [280]Iran University of Medical Sciences, Tehran, Iran; [281]Students' Scientific Research Center, Tehran University of Medical Sciences, Tehran, Iran; [282]Neuro Musculoskeletal Research Center, Iran University of Medical Sciences, Tehran, Iran; [283]Department of Public Health, Jordan University of Science and Technology, Irbid, Jordan; [284]Amity Institute of Forensic Sciences, Amity University, Noida, India; [285]Family Medicine Department, United Arab Emirates University, Al Ain, United Arab Emirates; [286]Primary Care Department, NHS North West London, London, UK; [287]Department of Critical Care Medicine, St. Luke's Aurora

Medical Center, Milwaukee, WI, USA; [288]Faculty of Nursing, Jerash University, Jerash, Jordan; [289]Department of Basic Medical Sciences, Yarmouk University, Irbid, Jordan; [290]School of Medicine, Kurdistan University of Medical Sciences, Sanandaj, Iran; [291]Department of Neurosurgery, Shahid Beheshti University of Medical Sciences, Tehran, Iran; [292]Bone and Joint Reconstruction Research Center, Iran University of Medical Sciences, Tehran, Iran; [293]Department of Public Health, New Mexico State University, Las Cruces, NM, USA; [294]Department of Cognition and Neuroscience, University of Texas, Dallas, TX, USA; [295]School of Health Sciences, Kristiania University College, Oslo, Norway; [296]Department of International Health and Sustainable Development, Tulane University, New Orleans, LA, USA; [297]Dpeartment of Public Health, Wachemo University, Addis Ababa, Ethiopia; [298]Department of Preventive and Social Medicine, University of Otago, Dunedin, New Zealand; [299]Social Determinants of Health Research Center, Shahid Beheshti University of Medical Sciences, Tehran, Iran; [300]Social Determinants of Health Research Center, Saveh University of Medical Sciences, Saveh, Iran; [301]Department of General Practice Family Medicine, Kharkiv National Medical University, Kharkiv, Ukraine; [302]Department of Epidemiology, IQVIA, Frankfurt, Germany; [303]Department of Gynecology, Philipps-Universität Marburg, Marburg, Germany; [304]Department of Biochemistry, All India Institute of Medical Sciences, Bhopal, India; [305]San Juan de Dios Sanitary Park, Barcelona, Spain; [306]Department of Anthropology, Panjab University, Chandigarh, India; [307]Department of Biochemistry, College of Medicine, University of Hail, Hail, Saudi Arabia; [308]Department of Orthopaedics, Medanta Hospital, Lucknow, India; [309]Faculty of Medicine and Health Science, Universitas Kristen Satya Wacana, Salatiga, Indonesia; [310]Nursing School, Taipei Medical University, Taipei, Taiwan; [311]Department of Nursing Science, Bayero University Kano, Kano, Nigeria; [312]Department of Health Policy and Strategy, Foundation for People-centric Health Systems, New Delhi, India; [313]SD Gupta School of Public Health, Indian Institute of Health Management Research University, Jaipur, India; [314]Department of Physiotherapy, Universitas Aisyiyah Yogyakarta, Yogyakarta, Indonesia; [315]Institute of Allied Health Sciences, National Cheng Kung University, Tainan, Taiwan;

[316]Department of Public Health, University of Helsinki, Helsinki, Finland; [317]Unit of Genetics and Public Health, Institute of Medical Sciences, Las Tablas, Panama; [318]Ministry of Health, Herrera, Panama; [319]Department of Otorhinolaryngology, Father Muller Medical College, Mangalore, India; [320]Clinical Pharmacy and Pharmacy Management, Kaduna State University, Kaduna, Nigeria; [321]University of Medicine and Pharmacy at Ho Chi Minh City, Ho Chi Minh City, Viet Nam; [322]Independent Consultant, Ho Chi Minh City, Viet Nam; [323]Department of Medical Humanities and Social Medicine, Ajou University School of Medicine, Suwon, South Korea; [324]Medial Research Collaborating Center, Ajou University Medical Center, Suwon, South Korea; [325]Department of Internal Medicine, University of Texas, Galveston, TX, USA; [326]Department of Preventive Medicine, Korea University, Seoul, South Korea; [327]School of Public Health, Dilla University, Dilla, Ethiopia; [328]School of Health Sciences, Western Sydney University, Campbelltown, NSW, Australia; [329]College of Medicine and Public Health, Flinders University, Bedford Park, SA, Australia; [330]School of Medicine, Federal University of Juiz de Fora, Juiz de Fora, Brazil; [331]Centre for Public Health and Wellbeing, University of the West of England, Bristol, UK; [332]Department of Physical Medicine and Rehabilitation, Tehran University of Medical Sciences, Tehran, Iran; [333]Department of Pediatric Cardiology, Tehran University of Medical Sciences, Tehran, Iran; [334]Department of Neurosurgery, University of Toronto, Toronto, ON , Canada; [335]Rabigh Faculty of Medicine, King Abdulaziz University, Jeddah, Saudi Arabia; [336]University Institute of Public Health, The University of Lahore, Lahore, Pakistan; [337]School of Medicine and Surgery, University of Milan Bicocca, Monza, Italy; [338]Laboratory of Public Health, Instituto Auxologico Italiano IRCCS (Italian Auxological Institute), Milan, Italy; [339]Department of Anatomy, Genetics and Biomedical Informatics, University of Colombo, Colombo, Sri Lanka; [340]Australian Regenerative Medicine Institute, Monash University, Clayton, VIC, Australia; [341]Department of Biomedical and Neuromotor Sciences, University of Bologna, Bologna, Italy; [342]Orthopedic Trauma Pathology Department, IRCCS, Bologna, Italy; [343]Australian Centre for Health Services Innovation, Queensland University of Technology, Kelvin Grove, QLD, Australia; [344]Digital Health and

Informatics Directorate, Queensland Health, Brisbane, QLD, Australia; [345]Department of Public Health, Debre Markos University, Debre Markos, Ethiopia; [346]Faculty of Medicine, Menoufia University, Shebin El-Kom, Egypt; [347]University Centre Varazdin, University North, Varazdin, Croatia; [348]Pacific Institute for Research & Evaluation, Calverton, MD, USA; [349]School of Public Health, Curtin University, Perth, WA, Australia; [350]International Ph.D. Program in Medicine, Taipei Medical University, Taipei, Taiwan; [351]Research Center for Artificial Intelligence in Medicine, Taipei Medical University, Taipei, Taiwan; [352]Department of Neurological Surgery, Dabat Health and Demograraphic Surveillance System, Qeshm, Iran; [353]Department of Neurological Surgery, Tabriz University of Medical Sciences, Tehran, Iran; [354]Internal Medicine Programme, Kyrgyz State Medical Academy, Bishkek, Kyrgyzstan; [355]Department of Atherosclerosis and Coronary Heart Disease, National Center of Cardiology and Internal Disease, Bishkek, Kyrgyzstan; [356]National Data Management Center for Health, Ethiopian Public Health Institute, Addis Ababa, Ethiopia; [357]Department of Health Education and Promotion, Ahvaz Jundishapur University of Medical Sciences, Ahvaz, Iran; [358]Digestive Diseases Research Institute, Tehran University of Medical Sciences, Tehran, Iran; [359]Molecular Biology Unit, Sirius Training and Research Centre, Khartoum, Sudan; [360]Bio-Statistical and Molecular Biology Department, Sirius Training and Research Centre, Khartoum, Sudan; [361]Faculty of Medicine, Tehran University of Medical Sciences, Tehran, Iran; [362]School of Medicine, Tehran University of Medical Sciences, Tehran, Iran; [363]Department of Pharmacy, Madda Walabu University, Bale Robe, Ethiopia; [364]Department of Pharmacology, Abadan School of Medical Sciences, Abadan, Iran; [365]Clinical Epidemiology and Public Health Research Unit, Burlo Garofolo Institute for Maternal and Child Health, Trieste, Italy; [366]School of Medicine, Shahid Beheshti University of Medical Sciences, Tehran, Iran; [367]Department of Epidemiology and Biostatistics, Kurdistan University of Medical Sciences, Sanandaj, Iran; [368]Division of Plastic and Reconstructive Surgery, University of Washington, Seattle, WA, USA; [369]Department of Medicine, Stanford University, Palo Alto, CA, USA; [370]Stanford Cardiovascular Institute, Stanford University, Palo Alto, CA, USA; [371]Non-communicable Disease

Research Center, Tehran University of Medical Sciences, Tehran, Iran; [372]Department of Community Medicine, Tabriz University of Medical Sciences, Tabriz, Iran; [373]Epidemiology Department, Aging Research Institute, Tabriz, Iran; [374]Department of Medicine, Democritus University of Thrace, Alexandroupolis, Greece; [375]Clinical Epidemiology Research Unit, Mexican Institute of Social Security, Villa de Alvarez, Mexico; [376]Postgraduate in Medical Sciences, Universidad de Colima, Colima, Mexico; [377]Department of Pediatrics, Shaqra University, Shaqra, Saudi Arabia; [378]Department of Pediatrics & Pediatric Pulmonology, Institute of Mother & Child Care, Multan, Pakistan; [379]Department of Research Methodology, Orthopaedic Research Group, Coimbatore, India; [380]Department of Biotechnology, Karpagam Academy of Higher Education, Coimbatore, India; [381]College of Medicine and Public Health, Flinders University, Adelaide, SA, Australia; [382]Department of Engineering, Western Sydney University, Sydney, NSW, Australia; [383]Laboratory of Public Health Indicators Analysis and Health Digitalization, Moscow Institute of Physics and Technology, Dolgoprudny, Russia; [384]Experimental Surgery and Oncology Laboratory, Kursk State Medical University, Kursk, Russia; [385]Department of Physiotherapy, Tehran University of Medical Sciences, Tehran, Iran; [386]Mysore Medical College and Research Institute, Government Medical College, Mysore, India; [387]Department of Rehabilitation Sciences, McMaster University, Hamilton, ON , Canada; [388]Student Research Committee, Tabriz University of Medical Sciences, Tabriz, Iran; [389]Department of Dental Public Health, King Abdulaziz University, Jeddah, Saudi Arabia; [390]Department of Health Policy and Oral Epidemiology, Harvard University, Boston, MA, USA; [391]Department of Biotechnology, University of Central Punjab, Lahore, Pakistan; [392]Department of Health Promotion, Zahedan University of Medical Sciences, Zahedan, Iran; [393]Independent Consultant, Tehran, Iran; [394]Department of Internal Medicine, Ardabil University of Medical Science, Ardabil, Iran; [395]Department of Medical Laboratory Sciences, Adigrat University, Adigrat, Ethiopia; [396]Department of Epidemiology, Non-Communicable Diseases Research Center (NCDRC), Tehran, Iran; [397]Division of Cardiology, Massachusetts General Hospital, Boston, MA, USA; [398]Department of Medical Engineering, University of South Florida, Tampa, FL, USA;

[399]Faculty of Medicine, Duy Tan University, Da Nang, Viet Nam; [400]Institue for Research and Training in Medicine, Biology and Pharmacy, Duy Tan University, Da Nang, Viet Nam; [401]Cardiovascular Research Department, Methodist Hospital, Merrillville, IL, USA; [402]Department of Surgery, Danang Family Hospital, Danang, Viet Nam; [403]Department of General Medicine, University of Medicine and Pharmacy at Ho Chi Minh City, Ho Chi Minh City, Viet Nam; [404]International Islamic University Islamabad, Islamabad, Pakistan; [405]Department of Pediatrics, University of Jos, Jos, Nigeria; [406]Department of Pediatrics, Jos University Teaching Hospital, Jos, Nigeria; [407]Health Promotion Research Center, Zahedan University of Medical Sciences, Zahedan, Iran; [408]School of Pharmacy, University of the Western Cape, Cape Town, South Africa; [409]Department of Nursing Science, Bowen University, Iwo, Nigeria; [410]College of Animal Science and Technology, Henan University of Science and Technology, Henan, China; [411]Department of Pharmacotherapy and Pharmaceutical Care, Medical University of Warsaw, Warsaw, Poland; [412]Department of Medicine, University of Ibadan, Ibadan, Nigeria; [413]Department of Medicine, University College Hospital, Ibadan, Ibadan, Nigeria; [414]Department of Respiratory Medicine, Jagadguru Sri Shivarathreeswara University, Mysore, India; [415]Department of Forensic Medicine and Toxicology, Kasturba Medical College, Mangalore, Mangalore, India; [416]Department of Emergency Medicine, University of Thessaly, Larissa, Greece; [417]Department of Emergency Medicine, University of Bern, Bern, Switzerland; [418]Yonsei University College of Medicine, Seodaemun-gu, South Korea; [419]Global Health Governance Programme, University of Edinburgh, Edinburgh, UK; [420]School of Dentistry, University of Leeds, Leeds, UK; [421]College of Dental Medicine, Roseman University of Health Sciences, South Jordan, UT, USA; [422]Centre of Molecular Medicine and Diagnostics (COMManD), Saveetha University, Chennai, India; [423]Department of Genetics, Yale University, New Haven, CT, USA; [424]Clinical Research Department, IRCCS Fondazione Don Carlo Gnocchi, Milan, Italy; [425]Centre for Primary Health Care and Equity, University of New South Wales, Kensington, NSW, Australia; [426]Department of Biology, University of Bahrain, Sakir, Bahrain; [427]Department of Statistics and Econometrics, Bucharest University of Economic

Studies, Bucharest, Romania; [428]Facultad de Medicina, Universidad Diego Portales (Diego Portales University), Santiago, Chile; [429]School of Cardiovascular and Metabolic Health, University of Glasgow, Glasgow, UK; [430]Medical School, Pham Ngoc Thach University of Medicine, Ho Chi Minh City, Viet Nam; [431]Department of Pediatric Orthopedic Surgery, Hôpital Necker - Enfants Malades, Paris, France; [432]Department of Global Health and Social Medicine, Harvard University, Boston, MA, USA; [433]Department of Maternal and Child Nursing and Public Health, Federal University of Minas Gerais, Belo Horizonte, Brazil; [434]Department of Health Sciences, Cihan University Sulaimaniya, Sulaymaniyah, Iraq; [435]Cihan University Sulaimaniya Research Center (CUSRC), Sulaymaniyah, Iraq; [436]Department of Biostatistics and Epidemiology, Ahvaz Jundishapur University of Medical Sciences, Ahvaz, Iran; [437]Department of Epidemiology, Iran University of Medical Sciences, Tehran, Iran; [438]Manipal TATA Medical College, Manipal Academy of Higher Education, Manipal, India; [439]Department of Physical Education and Sport Sciences, Lorestan University, Khoramabad, Iran; [440]Department of Radiology, Loyola University Medical Center, Maywood, IL, USA; [441]Department of Community Medicine, Mahatma Gandhi Medical College and Research Institute, Puducherry, India; [442]College of Pharmaceutical Sciences, Andhra University, Visakhapatnam, India; [443]Department of Cardiology, Tehran University of Medical Sciences, Tehran, Iran; [444]University of Social Welfare and Rehabilitation Sciences, Tehran, Iran; [445]Immunology Department, Shahid Beheshti University of Medical Sciences, Tehran, Iran; [446]Department of Primary Care and Public Health, Imperial College London, London, UK; [447]Academic Public Health England, Public Health England, London, UK; [448]Department of Internal Medicine, Manipal Academy of Higher Education, Mangalore, India; [449]Department of Biological Sciences, King Abdulaziz University, Jeddah, Egypt; [450]Department of Protein Research, Research and Academic Institution, Alexandria, Egypt; [451]Endocrinology and Metabolism Research Institute, Tehran University of Medical Sciences, Tehran, Iran; [452]Network of Immunity in Infection, Malignancy and Autoimmunity (NIIMA), Universal Scientific Education and Research Network (USERN), Tehran, Iran; [453]School of Public Health, Kermanshah University of Medical

Sciences, Kermanshah, Iran; [454]Department of Public Health, Masaryk University, Brno, Czech Republic; [455]Czech National Centre for Evidence-based Healthcare and Knowledge Translation, Masaryk University, Brno, Czech Republic; [456]Department of Clinical Research, Federal University of Uberlândia, Uberlândia, Brazil; [457]Department of Physical Medicine and Rehabilitation, Shiraz University of Medical Sciences, Shiraz, Iran; [458]Burn and Wound Healing Research Center, Shiraz University of Medical Sciences, Shiraz, Iran; [459]Department of Labour, Directorate of Factories Government of West Bengal, Kolkata, India; [460]Advanced Campus Governador Valadares, Juiz de For a Federal University, Governador Valadares, Brazil; [461]Nursing Department, Universidade Presidente Antônio Carlos (President Antônio Carlos University), Governador Valadares, Brazil; [462]Cardiovascular Department, Zagazig University, Zagazig, Egypt; [463]Sharjah Institute for Medical Research, University of Sharjah, Sharjah, United Arab Emirates; [464]Center for Health Related Social and Behavioral Sciences Research, Shahroud University of Medical Sciences, Shahroud, Iran; [465]Multidisciplinary Laboratory Foundation University School of Health Sciences (FUSH), Foundation University, Islamabad, Pakistan; [466]International Center of Medical Sciences Research (ICMSR), Islamabad, Pakistan; [467]Connective Tissue Diseases Research Center, Tabriz University of Medical Sciences, Tabriz, Iran; [468]Department of Psychosocial Science, University of Bergen, Bergen, Norway; [469]Sharjah Institute of Medical Sciences, University of Sharjah, Sharjah, United Arab Emirates; [470]Applied Biomedical Research Center, Mashhad University of Medical Sciences, Mashhad, Iran; [471]Biotechnology Research Center, Mashhad University of Medical Sciences, Mashhad, Iran; [472]Ludwig Maximilian University of Munich, Munich, Germany; [473]Institute for Employment Research, Nuremberg, Germany; [474]Department of Oral and Maxillofacial Surgery, University College Hospital, Ibadan, Ibadan, Nigeria; [475]Campaign for Health and Neck Cancer Education (CHANCE) Programme, Cephas Health Research Initiative Inc, Ibadan, Nigeria; [476]Faculty of Pharmacy, Mansoura University, Mansoura, Egypt; [477]Mark and Mary Stevens Neuroimaging and Informatics Institute, University of Southern California, Los Angeles, CA, USA; [478]Department of Neurology, Charité University Medical Center

Berlin, Berlin, Germany; [479]Department of Neurology, University of Southern Denmark, Odense, Denmark; [480]School of Public Health, Taipei Medical University, Taipei, Taiwan; [481]Department of Anatomy, Ras Al Khaimah Medical and Health Sciences University, Ras Al Khaimah, United Arab Emirates; [482]Health Sciences Center, Federal University of Reconcavo of Bahia, Santo Antônio de Jesus, Brazil; [483]School of Public Health and Health Management, University of Belgrade, Belgrade, Serbia; [484]Research Development Coordination Section, Pakistan Health Research Council, Islamabad, Pakistan; [485]School of Sciences, University of Management and Technology, Lahore, Pakistan; [486]Department of Pharmacology and Research, All India Institute of Medical Sciences, Jodhpur, India; [487]Indira Gandhi Medical College and Research Institute, Puducherry, India; [488]Department of Paediatrics, University of Melbourne, Parkville, VIC, Australia; [489]Centre for Adolescent Health, Murdoch Childrens Research Institute, Parkville, VIC, Australia; [490]Department of Human Genetics and Molecular Medicine, Central University of Punjab, Bathinda, India; [491]Department of Medicine and Surgery, Government Doon Medical College, Dehradun, India; [492]National Heart, Lung, and Blood Institute, National Institute of Health, Rockville, MD, USA; [493]Department of Mechanical Engineering, Arak University of Technology, Arak, Iran; [494]Department of Neurology, Tehran University of Medical Sciences, Tehran, Iran; [495]Department of Medicine, Tehran University of Medical Sciences, Tehran, Iran; [496]Department of Neuroscience, University of Calgary, Calgary, AB, Canada; [497]Department of Neuroimmunulogy, Universal Scientific Research Network (USERN), Tehran, Iran; [498]Independent Consultant, Karachi, Pakistan; [499]Department of Pathobiology, Shahid Bahonar University of Kerman, Kerman, Iran; [500]Department of Clinical Review and Safety, Baim Institute for Clinical Research, Boston, MA, USA; [501]Beth Israel Deaconess Medical Center, Harvard University, Boston, MA, USA; [502]Department of Pharmaceutical Care, Tehran University of Medical Sciences, Tehran, Iran; [503]Research Center for Rational Use of Drugs, Tehran University of Medical Sciences, Tehran, Iran; [504]Nahavand School of Allied Medical Sciences, Hamadan University of Medical Sciences, Hamadan, Iran; [505]Dr Ram Manohar Lohia Hospital, New Delhi, India; [506]Faculty of Medicine and

Health, University of New South Wales, Sydney, NSW, Australia; [507]Department of Physiotherapy, Kathmandu University, Dhulikhel, Nepal; [508]National Institute of Infectious Diseases, Tokyo, Japan; [509]Finnish Institute of Occupational Health, Helsinki, Finland; [510]Clinical Immunology and Hematology, Sofiamed University Hospital, Sofia, Bulgaria; [511]Department of Genetics, Sofia University "St. Kliment Ohridiski", Sofia, Bulgaria; [512]Unit of Basic Medical Sciences, University of Khartoum, Khartoum, Sudan; [513]Department of Medical Microbiology and Infectious Diseases, Erasmus University, Rotterdam, Netherlands; [514]Department of Physical Therapy, Shiraz University of Medical Sciences, Shiraz, Iran; [515]School of Medicine, University of Alabama at Birmingham, Birmingham, AL, USA; [516]Medicine Service, US Department of Veterans Affairs (VA), Birmingham, AL, USA; [517]Department of Radiodiagnosis, All India Institute of Medical Sciences, Bathinda, India; [518]Department of Pharmacology, Adesh Institute of Medical Sciences and Research, Bathinda, India; [519]Maternal and Child Health Division, International Centre for Diarrhoeal Disease Research, Bangladesh, Dhaka, Bangladesh; [520]Department of Infectious Diseases and Epidemiology, Pirogov Russian National Research Medical University, Moscow, Russia; [521]Department of Pathology, Ross University School of Medicine, Bridgetown, Barbados; [522]Department of Pathology, American University of the Caribbean School of Medicine, Cupecoy, Saint Martin; [523]Department of Nursing, Dire Dawa University, Dire Dawa, Ethiopia; [524]Nutrition and Dietetics Department, Federal Research Institute of Nutrition, Biotechnology and Food Safety, Moscow, Russia; [525]Department of Internal Disease, Pirogov Russian National Research Medical University, Moscow, Russia; [526]Analytical and Applied Economics Department, Utkal University, Bhubaneswar, India; [527]School of Public Health and Preventive Medicine, Monash University, Melbourne, VIC, Australia; [528]Department of Economics, Rice University, Houston, TX, USA; [529]Department of Research and Innovation, Enventure Medical Innovation, Houston, TX, USA; [530]Pediatric Intensive Care Unit, King Saud University, Riyadh, Saudi Arabia; [531]Outpatient Department, Wollega University, Bedele town, Ethiopia; [532]Health Management Department, Ariel University, Ariel, Israel; [533]Rheumatology and Immunology Unit, Mansoura University, Mansoura, Egypt;

[534]Institute of Applied Health Research, University of Birmingham, Birmingham, UK; [535]Faculty of Medicine, University of Southampton, Southampton, UK; [536]Faculty of Public Health, Universitas Sam Ratulangi, Manado, Indonesia; [537]Saveetha Dental College and Hospitals, Saveetha Institute of Medical and Technical Sciences (SIMATS), Chennai, India; [538]SRM College of Pharmacy, SRM Institute of Science and Technology (SRMIST), Chennai, India; [539]Department of Epidemiology and Biostatistics, Haramaya University, Harar, Ethiopia; [540]Department of Pharmacology, All India Institute of Medical Sciences, Deoghar, India; [541]Department of Biosciences and Biotechnology, University of Medical Sciences, Ondo, Ondo, Nigeria; [542]Urmia University of Medical Sciences, Urmia, Iran; [543]Department of Cardiovascular Sciences, Katholieke Universiteit Leuven, Leuven, Belgium; [544]UKK Institute, Tampere, Finland; [545]Faculty of Medicine and Health Technology, Tampere University, Tampere, Finland; [546]Raffles Neuroscience Centre, Raffles Hospital, Singapore, Singapore; [547]Yong Loo Lin School of Medicine, National University of Singapore, Singapore, Singapore; [548]Department of Health Care Administration and Economics, National Research University Higher School of Economics, Moscow, Russia; [549]Institute for Global Health Innovations, Duy Tan University, Da Nang, Viet Nam; [550]Department of Psychiatry, University of São Paulo, São Paulo, Brazil; [551]Department of Physical Therapy, Naresuan University, Phitsanulok, Thailand; [552]Department of Community Medicine, Rajarata University of Sri Lanka, Anuradhapura, Sri Lanka; [553]Institute of Health and Society, University of Oslo, Oslo, Norway; [554]Department of Neurology, Technical University of Munich, Munich, Germany; [555]Department of Orthopaedics, Wenzhou Medical University, Wenzhou, China; [556]Psychology Department, University of Sheffield, Sheffield, UK; [557]Department of Microbiology and Immunology, Zagazig University, Zagazig, Egypt; [558]Department of Cells and Tissues, Molecular Biology Institute of Barcelona, Barcelona, Spain; [559]Department of Neuropsychopharmacology, National Center of Neurology and Psychiatry, Kodaira, Japan; [560]Department of Public Health, Juntendo University, Tokyo, Japan; [561]Macquarie Medical School, Macquarie University, Sydney, NSW, Australia; [562]Department of Health Policy and Management, Jackson State University, Jackson,

MS, USA; [563]School of Business & Economics, Universiti Putra Malaysia (University of Putra Malaysia), Kuala Lumpur, Malaysia; [564]Faculty of Medicine and Health Sciences, Hodeidah University, Hodeidah, Yemen; [565]Department of Virology, University of Helsinki, Helsinki, Finland; [566]Jockey Club School of Public Health and Primary Care, The Chinese University of Hong Kong, Hong Kong, China; [567]Department of Orthopaedics, Shandong University, Jinan, China; [568]General Practice, Erasmus University Medical Center, Rotterdam, Netherlands; [569]Department of Biochemistry and Pharmacogenomics, Medical University of Warsaw, Warsaw, Poland; [570]Department of Anatomy, Addis Ababa University, Addis Ababa, Ethiopia; [571]School of Public Health Sciences, University of Waterloo, Waterloo, ON, Canada; [572]College of Medicine, Sulaiman Alrajhi University, Al Bukairiyah, Saudi Arabia; [573]Department of Nursing, Yasuj University of Medical Sciences, Yasuj, Iran; [574]Department of Rheumatology and Immunology, Sichuan Provincial People's Hospital, School of Medicine, University of Electronic Science and Technology of China, Chengdu, China
